# Taking Advantage of a Luminescent ESIPT-Based Zr-MOF for Fluorochromic Detection of Multiple External Stimuli: Acid and Base Vapors, Mechanical Compression, and Temperature

**DOI:** 10.1021/acsami.3c14348

**Published:** 2023-11-20

**Authors:** Francisco Sánchez, Mario Gutiérrez, Abderrazzak Douhal

**Affiliations:** Departamento de Química Física, Facultad de Ciencias Ambientales y Bioquímica, INAMOL, Universidad de Castilla-La Mancha, Avenida Carlos III, S/N, 45071 Toledo, Spain

**Keywords:** Luminescent MOF, Photodynamics, Vapoluminescent Detection, Fluorochromic Compression Response, Thermoluminescent Response

## Abstract

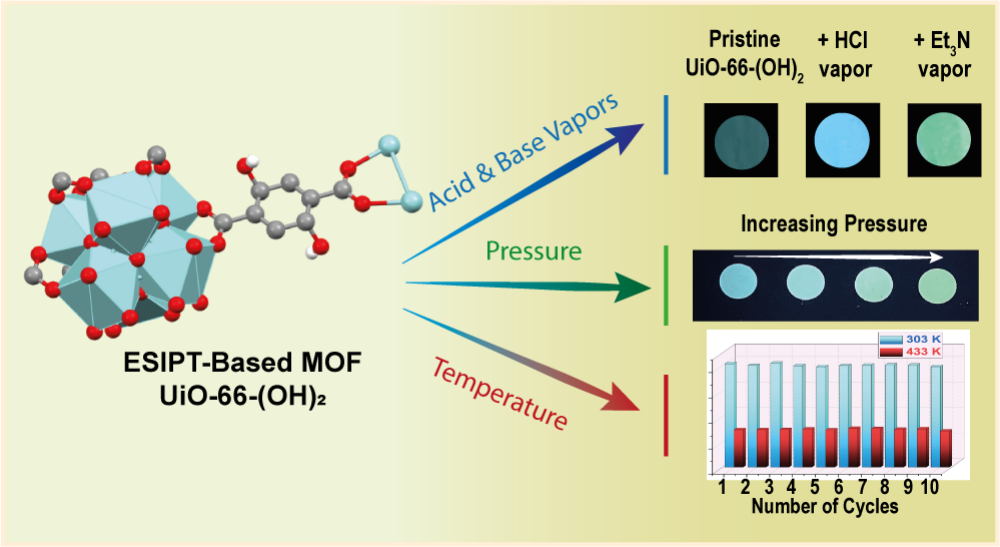

Luminescent materials responsive to external stimuli have captivated great attention owing to their potential implementation in noninvasive photonic sensors. Luminescent metal–organic frameworks (LMOFs), a type of porous crystalline material, have emerged as one of the most promising candidates for these applications. Moreover, LMOFs constructed with organic linkers that undergo excited-state intramolecular proton-transfer (ESIPT) reactions are particularly relevant since changes in the surrounding environment induce modifications in their emission properties. Herein, an ESIPT-based LMOF, UiO-66-(OH)_2_, has been synthesized, spectroscopically and photodynamically characterized, and tested for detecting multiple external stimuli. First, the spectroscopic and photodynamic characterization of the organic linker (2,5-dihydroxyterephthalic acid (DHT)) and the UiO-66-(OH)_2_ MOF demonstrates that the emission properties are mainly governed by the enol → keto tautomerization, occurring in the organic linker via the ESIPT reaction. Afterward, the UiO-66-(OH)_2_ MOF proves for the first time to be a promising candidate to detect vapors of acid (HCl) and base (Et_3_N) toxic chemicals, changes in the mechanical compression (exercised pressure), and changes in the temperature. These results shed light on the potential of ESIPT-based LMOFs to be implemented in the development of advanced optical materials and luminescent sensors.

## 1 Introduction

Nowadays, there exists an increasing demand on the design and developing of novel advanced materials with the ability to respond to external stimuli, owing to their potential implementation in multiple types of noninvasive sensors.^1,2^ These sensors can be exploited in a number of industrial processes, working/housing environments, or healthcare purposes by detecting toxic chemicals (e.g., harmful volatile compounds, chemical pollutants, etc.), biological markers, or physical stimuli (e.g., temperature, pressure).^3,4^ Among all the possibilities, luminescent-based sensors are one of the most promising since they fulfill most of the characteristics desired for a sensor: (i) high sensibility and selectivity; (ii) can be constructed to be portable; (iii) low cost of fabrication; and (iv) easy to use. In this sense, the seeking of novel luminescent materials able to respond to multiple external stimuli has become a priority for several interdisciplinary researchers. One of the most popular materials over the past years is luminescent metal–organic frameworks (LMOFs), a type of porous crystalline material formed by the interlinked connection of metal ions or clusters and organic linkers.^5−7^ LMOFs have demonstrated a great potential in a number of applications such as chemical detection,^8−12^ luminescent thermometers,^13−15^ mechanical compression detection,^3,16,17^ or optoelectronic devices,^16,18,19^ to cite few of them.

Moreover, luminescent materials with the ability of transferring a hydrogen (H) atom from a donor moiety to an acceptor one upon photoexcitation (a process known as excited-state intramolecular proton transfer, ESIPT) are also excellent candidates for detecting changes in the surrounding environment,^20,21^ since the modulation of the ESIPT reaction induces changes in the luminescent properties of the material, and their large Stokes shift minimizes or prevents undesired photophysical phenomena such as autoabsorption.^22,23^ Hence, the combination of MOFs and ESIPT linkers is a great opportunity to develop advanced LMOF materials capable of detecting multiple external stimuli. The ESIPT process in MOFs can occur either as a result of an ESIPT reaction in the organic linkers^24−26^ or in ESIPT molecules encapsulated within the MOF pores.^10^ In this sense, one of the most commonly used organic linkers for the fabrication of ESIPT-based LMOFs is 2,5-dihydroxyterephthalic acid (DHT).^27−30^ This linker can emit light from the enol (blue color) or keto (green-yellowish color) tautomers when it is part of the MOF network, and the ultimate emission color of the material depends on the efficiency of the ESIPT reaction to form the keto species.^28,29^ For example, it has been reported that depending on the metal used in the synthesis, the DHT-based MOFs had different crystalline structures, affecting the ESIPT reaction and, therefore, modifying the final emission color of the material.^29^ Most of the DHT-based MOFs have been employed to detect traces of water, different ions, and harmful chemical compounds in solutions.^27−29,31−39^ However, their use in the detection of volatile compounds is more scarce.^37,40,41^ To the best of our knowledge, there are no examples of luminescent UiO-66-(OH)_2_ used for specific detection of vapors of base and acid compounds nor external physical stimuli (e.g., changes in temperature or pressure).

Hence, herein, we have synthesized an ESIPT-based LMOF, UiO-66-(OH)_2_, to leverage the ESIPT reaction in the organic linker, aiming to explore its capacity to detect vapors of acid (HCl) and base (Et_3_N) chemicals as well as other external stimuli such as mechanical compression and temperature. To this end, we have first characterized the spectroscopic and photodynamics properties of the DHT molecule in different solutions to unveil the ESIPT reaction and the emission characteristics of the enol and keto tautomers, as well as other possible conformers (i.e., anions). Then, we have explored the spectroscopic and photodynamic properties of the UiO-66-(OH)_2_ MOF, both in suspension and in the solid state (powder form), and compared the results to those found for DHT in solutions. We demonstrate that the emission of the MOF is mainly originated by the organic linker, showing two emission bands ascribed to the enol (∼465 nm) and keto (∼525 nm) tautomeric species. Afterward, the UiO-66-(OH)_2_ powder material was used to detect the presence of vapors of acid (HCl) and base (Et_3_N) compounds. The MOF exhibits a clear turn-on and wavelength-shifted (emission color change) response, as a consequence of the large difference between the emission of the enol and keto tautomers. The ability of the UiO-66-(OH)_2_ MOF to detect changes in the mechanical compression (pressure) and temperature was also tested. Upon different applied pressures, the luminescence of the MOF shifts from blue to green as a result of an increase in the population of the keto tautomer. Moreover, high temperatures (up to 433 K) induce quenching in the emission intensity of UiO-66-(OH)_2_. The luminescent response of this MOF toward the increment in temperature is lineal and reproducible. Therefore, our results reflect the potential of this proton-transfer MOF to be deployed in four different types of luminescent sensors, specifically for vapors of acids and bases, temperature, and pressure. Up until now, there have been no reported examples of other ESIPT-based MOFs showing this sensing versatility (four different external stimuli sensing), and this might open novel avenues for developing more efficient ESIPT-based LMOF materials and related luminescent sensors.

## 2 Experimental Section and Methods

### 2.1 Materials

Zirconium(IV) chloride anhydrous (ZrCl_4_, 98%) was purchased from Acros Organics. 2,5-Dihydroxyterephthalic acid (DHT, 98%) was acquired from Sigma-Aldrich (Merck). N,N-Dimethylformamide (Essents, 99.5%), triethylamine (99%), and hydrochloric acid (HCl, 37%) were acquired from Scharlau Company. All of the chemicals were used as received.

### 2.2 Synthesis and Characterization of UiO-66-(OH)_2_

UiO-66-(OH)_2_ was synthesized following the procedure previously described for UiO-66, with some modifications.^42^ Briefly, 1.6 mmol of ZrCl_4_, 1.6 mmol of 2,5-dihydroxyterephthalic acid (DHT), 2 mL of hydrochloric acid (HCl), and 10 mL of N,N-dimethylformamide (DMF) were poured into a Pyrex bottle and heated to 120 °C for 24 h. Afterward, the obtained pale-yellow powder was collected by centrifugation (10 000 rpm, 10 min) and thoroughly washed with DMF (∼20 cycles of washing and centrifuging) to be rid of the unreacted linker. Finally, the UiO-66-(OH)_2_ material was dried at 110 °C for 24 h, collected, and kept in a desiccator under a vacuum.

### 2.3 Methods and Materials Characterization

The steady-state UV–visible absorption/reflectance and fluorescence spectra of the samples have been recorded using Jasco V-670 and FluoroMax-4 (Jobin-Yvon) spectrophotometers, respectively. For the UiO-66-(OH)_2_, the UV–visible diffuse reflectance spectra were recorded using a 60 nm integrating sphere, ISN-723. The obtained signals were converted to the Kubelka–Munk function *F* = ((1 – *R*)^2^/2*R*), where *R* is the diffuse reflectance from the sample.

The X-ray diffraction patterns of polycrystalline MOF powder were obtained by using a Bruker D8 Advance instrument with a Bragg–Brentano geometry and a silica layer LyNXEYE XE detector. The conditions used were Cu Kα radiation, a range of measurement between 2 and 35° (2θ) with a step of 0.05°, and a dwell time of 1.5 s per step.

The Fourier-transformed infrared (FTIR) spectra were recorded using a PerkinElmer Spectrum 100 equipped with Mid-IR deuterated triglycine sulfate and a mercury cadmium telluride detector and with universal attenuated total reflection.

The SEM images of the UiO-66-(OH)_2_ MOF were obtained by using a field emission scanning electron microscope (SEM, Zeiss GeminiSEM 500, Oberkochen, Germany) operating in high vacuum mode. The MOF sample was properly placed and mounted onto standard aluminum SEM stubs using conductive carbon adhesive tabs and coated with gold.

N_2_ adsorption and desorption isotherm measurements were performed on a NOVAtouch LX2, Quantachrome Instruments brand. Nitrogen gas was used as an adsorbate, and the temperature at which the tests were performed was the temperature of liquid nitrogen (77 K). For this test, the sample was degassed prior to the adsorption measurements at 100 °C for 24 h.

Elemental analysis of UiO-66-(OH)_2_ was performed using a LECO CHNS-932 microelemental analyzer. The sample is analyzed using the Dumas method, which involves oxidative combustion in a pure oxygen atmosphere at a high temperature. Approximately 1 mg of the sample is weighed for each measurement using a microbalance with six decimal places (METTLER-TOLEDO XP6) and encapsulated in a tin capsule prior to analysis. The working temperature of the elemental analyzer is 1000 °C, and pure oxygen doses are added fractionally to ensure complete combustion of the sample.

Picosecond (ps) emission decays were collected using a time-correlated single photon counting (TCSPC) system. The samples were pumped by a 40 ps-pulsed (<1 mW, 40 MHz repetition rate) diode-laser (PicoQuant) centered at 371 nm. The instrumental response function (IRF) of the system is around ∼70 ps. The fluorescence signal was gated at the magic angle (54.7°) and monitored at a 90° angle with respect to the excitation beam at discrete emission wavelengths. The decays were deconvoluted and fitted to a multiexponential function using the FLUOFIT package (PicoQuant). The quality of the fit was estimated considering the value of χ^2^, which was always below 1.2, and the distribution of the residues. All of the experiments were performed at 293 K.

For the vapoluminescent detection experiments, 2 mg of UiO-66-(OH)_2_ MOF powder was deposited and homogeneously spread on a paper strip with a spatula in a total surface of 1.8 × 1.1 cm^2^. This surface was selected to ensure that the MOF material is covering all of the irradiation area (much smaller than 1.8 × 1.1 cm^2^), so the MOF is evenly irradiated, and the possible dispersion of light by the paper strip is eliminated all at once. The paper strip was placed on a container with saturated atmospheres of HCl or Et_3_N for different periods of time. The emission of the sample was collected before and after its exposure to the corresponding vapors of acid (HCl) or base (Et_3_N). Furthermore, we have compared the emission spectra of the UiO-66-(OH)_2_ MOF deposited on the paper strip with those of the pristine powder and obtained the same shape and intensity of the emission spectra, indicating that there are no interferences coming from the paper itself.

For the mechanoluminescent response experiments, we prepared four different pellets using 120 mg of MOF powder for each sample. The MOF powder was introduced in a holder with a diameter of 1.3 cm, and then it was compressed under a nominal stress of 1, 2, 4, and 10 tons, using a Specac Atlas 15 Ton Hydraulic Press. The emission of the pellets was measured and compared with that of the MOF powder.

For the thermochromic response experiments, ∼200 mg of UiO-66-(OH)_2_ MOF powder was placed in a metal holder coupled to a PTC heating plate. A proportional–integral–derivative (PID) controller is used to measure and control the temperature during the experiment. The material was kept at each temperature for 10 min before measuring the emission spectrum to ensure the thermal stabilization.

## 3 Results and Discussion

### 3.1 Brief Summary of the UV–Vis Steady-State and Time-Resolved Photophysical Characterization of DHT Linker in DMF Solution

**Scheme 1:**
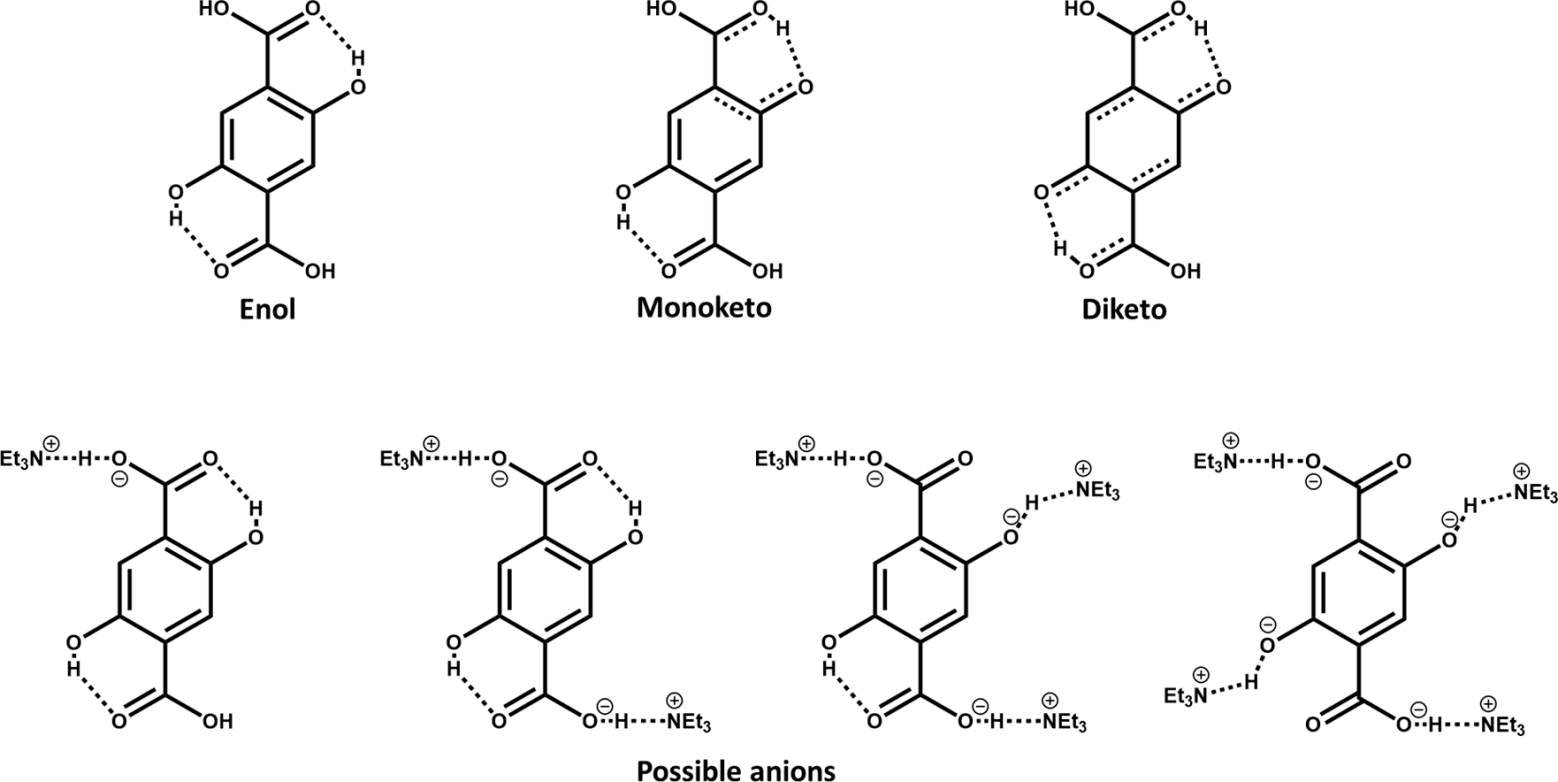
Molecular Structures of Different Possible Conformers and Anions of 2,5-Dihydroxyterephthalic Acid (DHT) When Complexed with Triethylamine

To better understand the spectroscopic and photophysical properties of UiO-66-(OH)_2_, we have first investigated those of the organic linker in DMF solution. The detailed results are provided in the Supporting Information (SI, section 1), while here we just describe the most noticeable findings. We demonstrate that the DHT linker might exist under different species, as depicted in Scheme 1, with each of them showing different spectroscopic properties. In brief, when the linker is dissolved in pure DMF, it shows a single absorption band with its intensity maximum at 370 nm, due to the absorption of the enol species most probably having double intramolecular H bonds (Scheme 1). The emission spectrum shows a large Stokes shift (7980 cm^–1^) with an intensity maximum at 525 nm, and it corresponds to the fluorescence of the keto tautomers formed after an excited-state intramolecular proton-transfer (ESIPT) reaction in the absorbing enol forms. When the linker is dissolved in a mixture of DMF and HCl, the absorption spectrum remains comparable to the one found in pure DMF, however the emission spectrum exhibits two emission bands with intensity maxima at 450 nm (emission of the enol tautomer) and 525 nm (emission of the keto species), respectively. The presence of HCl induces the emission of the enol form since it might partially protonate the C=O groups of the carboxylic acids of DHT molecules, hindering the ESIPT reaction. Interestingly, when the DHT linker is dissolved in DMF containing Et_3_N, the absorption spectrum is shifted toward shorter wavelengths (355 nm), while the emission band is very broad and red-shifted, with a maximum intensity at 570 nm. This suggests that the emission in this mixture mainly arises from anionic species. These results corroborate the possible existence of multiple species of DHT linker upon protonation or deprotonation, as depicted in Scheme 1.

### 3.2 Structural and Chemical Characterization of UiO-66-(OH)_2_

The crystalline structure of UiO-66-(OH)_2_ was investigated by powder X-ray diffraction (PXRD) measurements (Figure S3A). The PXRD pattern of UiO-66-(OH)_2_ coincides with that of UiO-66, indicating an isostructural topology and confirming the crystallinity of our material.^43^

The FTIR spectrum of the UiO-66-(OH)_2_ resembles that previously published for this material (Figure S3B).^44−46^ Briefly, some of the most noticeable peaks are those between 500 and 800 cm^–1^, corresponding to Zr–O stretching, bending, or twisting modes; a peak at 660 cm^–1^ ascribed to μ3-O stretching mode; a peak around 1200 cm^–1^, assigned to the phenolic C–OH stretching vibration; the band at 1380 cm^–1^ corresponding to the C–C ring; the bands at 1450 and 1650 cm^–1^ attributed to the OCO symmetric and asymmetric stretching modes, respectively; and the broad band spanning from ∼3300–2800 cm^–1^ ascribed to the – OH stretching vibrational mode.^47^ Both the PXRD and FTIR data prove the success in the synthesis of UiO-66-(OH)_2_ MOF material.

The morphology and crystal size of the UiO-66-(OH)_2_ MOF have also been characterized by SEM. The images in Figure S4 reveal that the MOF crystals have a particle size around 200–300 nm, with some crystals showing a triangular-base pyramid shape and most of them showing a nondefined morphology. Similar results have been previously reported for this MOF.^29,48^

To investigate the porosity of this MOF material and the plausible existence of defects, we measured the N_2_ BET isotherm (Figure S5) and elemental analysis composition. The N_2_ BET surface area of our synthesized UiO-66-(OH)_2_ MOF is 560.2 m^2^/g, comparable to that previously reported (560 m^2^/g) for UiO-66-(OH)_2_ synthesized under the same experimental conditions.^43^ This value is larger than that theoretically calculated (400 m^2^/g), and the difference has been attributed to the absence of linkers in this MOF due to the use of HCl in the synthesis.^43^ On the other hand, we have also performed elemental analysis of the synthesized UiO-66-(OH)_2_ and found the following composition: C, 29.4%; H, 3.3%; and N, 2.4%. The presence of N suggests the existence of DMF molecules in the MOF structure, something expected since the material is dried at 110 °C. We estimated that there exist around 3.5 molecules of DMF per molecular formula: Zr_6_O_4_(OH)_4_[C_6_H_2_(OH)_2_(COO)_2_]_6_(DMF)_3.5_. For this molecular formula, the calculated elemental composition is C, 33.3%; H, 2.5%; and N, 2.3%. Hence, the experimental value obtained for C, 29.4%, is less than the theoretical one (33.3%), suggesting the presence of missing-linker defects, in agreement with the N_2_ BET results.

### 3.3 UV–Visible Steady-State Properties of UiO-66-(OH)_2_ in a DMF Suspension

**Figure 1:**
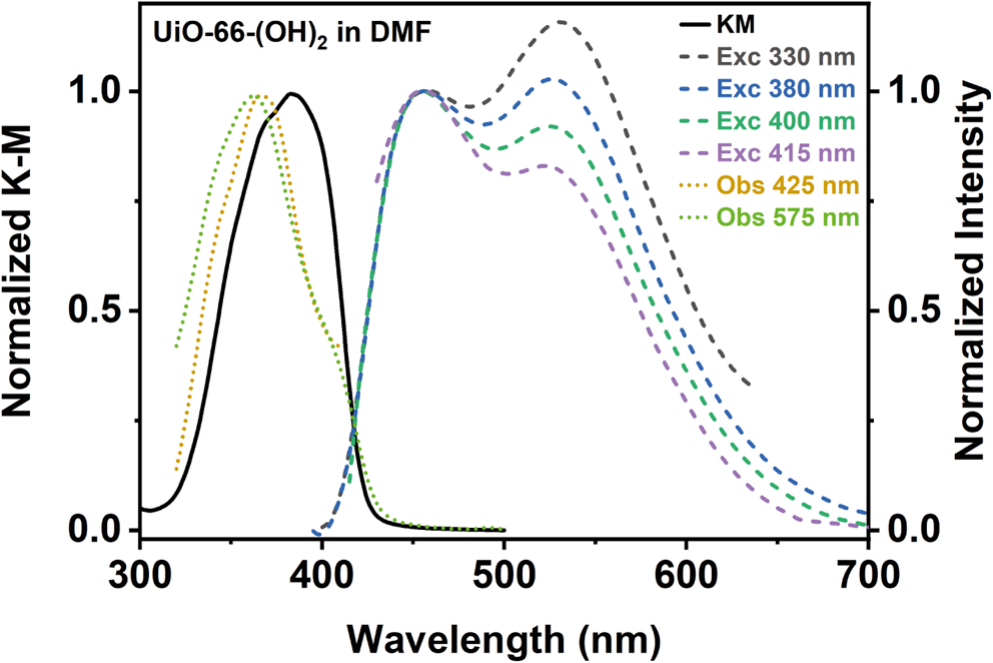
Normalized diffuse reflectance (converted to the K-M function, black solid line), excitation spectra (dotted lines), and normalized (to the blue emission band intensity) emission spectra (dashed lines) of UiO-66-(OH)_2_ in a DMF suspension. The emission spectra are normalized to the maximum of the enol emission (465 nm). The different excitation and observation wavelengths are indicated in the inset.

To unveil the spectroscopic properties of UiO-66-(OH)_2_, we have first characterized the UV–vis optical behavior in a DMF suspension and compared it with that of the linker described in the previous section. The absorption spectrum of UiO-66-(OH)_2_ in DMF suspension is a band with its intensity maximum at ∼385 nm and a shoulder around 370 nm (Figure 1). The shift in the absorption maximum of the MOF toward longer wavelengths when compared to the pristine DHT linker (Figure S6A) reflects a charge transfer interaction between the organic linker and the Zr-metal clusters, as previously described for other Zr-based MOFs.^49−52^ On the other hand, the emission spectrum of UiO-66-(OH)_2_ in a DMF suspension is composed of two bands having intensity maxima at ∼465 and ∼525 nm. In agreement with our previous discussion, these emission bands correspond to the emission of the enol (465 nm) and keto (525 nm) tautomers of the DHT linker in the MOF. When the DHT linker is taking part in the MOF structure, the carboxylate groups are anchored to the Zr metal atoms, because the ESIPT reaction is less favored, and therefore, the enol species can emit light. The observation of two emission bands corresponding to enol and keto tautomers of DHT linkers have been previously described for other DHT-based MOFs.^27,28,31^ Moreover, we have observed that the relative intensity ratio between these two emission bands depends on the excitation wavelength (Figure 1). When the UiO-66-(OH)_2_ MOF is photoexcited with higher energies (lower wavelengths), the emission of the keto tautomer (525 nm) is enhanced. This is explained considering that higher photoexcitation energies will populate higher electronic or vibrational states of the DHT linker, favoring the surpassing of the ESIPT energy barrier and leading to the formation of a larger population of keto tautomers and, therefore, less emission from the enol forms.

As shown in Figure 1, the excitation spectra collected at the bluest (425 nm) and reddest (575 nm) regions are comparable. Moreover, the excitation spectra are similar to the absorption one (with some changes in the ratio between the 370 and 385 nm peaks), indicating a common origin of the excited species, similar to what we observed for the pristine linker in DMF.

### 3.4 UV–Vis Steady-State and Time-Resolved Photophysical Studies of UiO-66-(OH)_2_ in the Solid State

**Figure 2:**
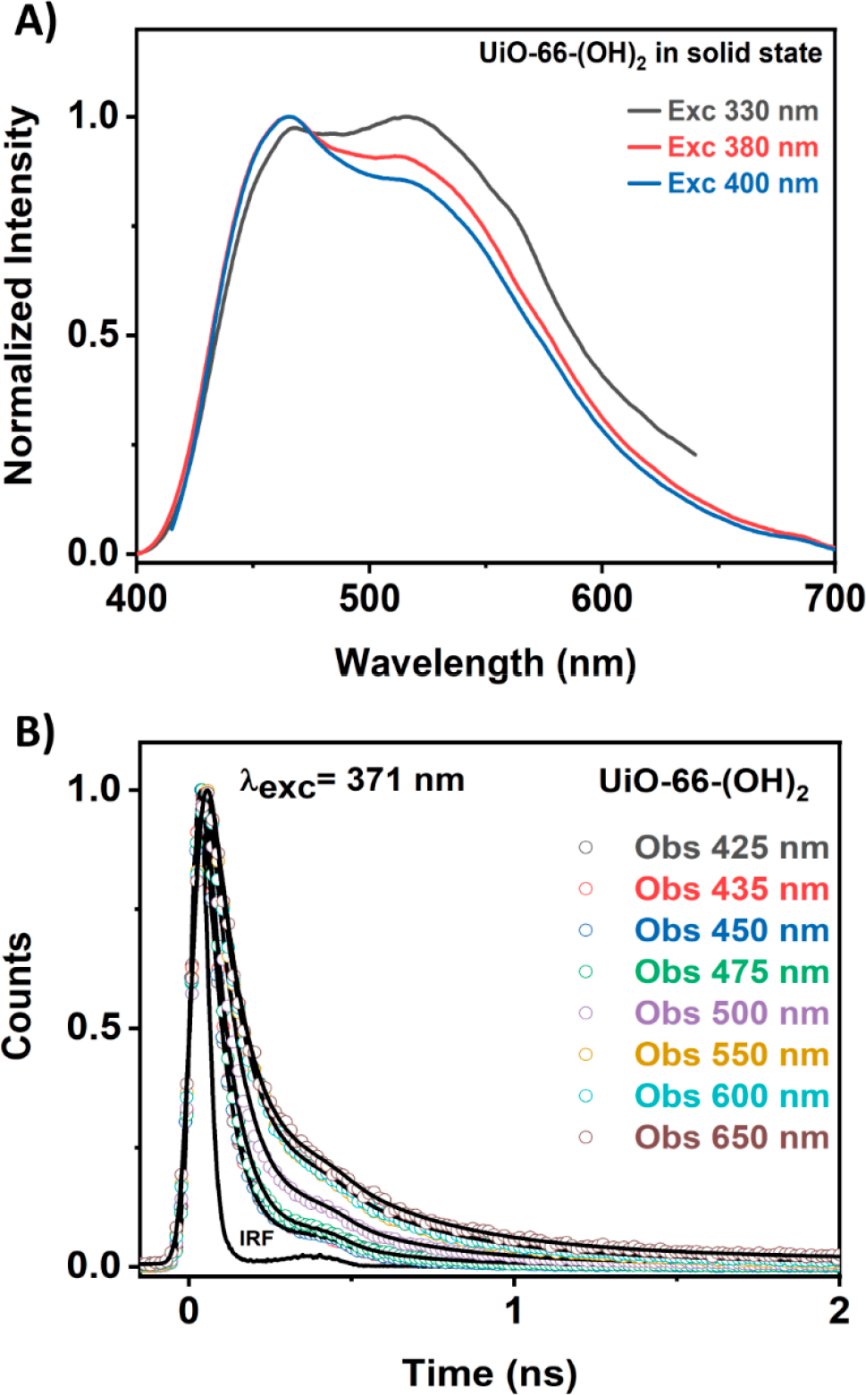
(A) Normalized emission spectra of UiO-66-(OH)_2_ in powder form at different excitation wavelengths (indicated in the figure). (B) Magic-angle emission decays of UiO-66-(OH)_2_ in powder form. The sample was excited with a 371 nm pulsed laser and probed at the indicated wavelengths. The solid black lines correspond to the fits of the decays using a multiexponential function, while the IRF is the instrumental response function.

Once the spectroscopic properties of UiO-66-(OH)_2_ in a DMF suspension were deciphered, we were interested in unravelling the spectroscopic and photodynamics behavior of this MOF in the solid state (powder form), as most of the luminescent materials used for detecting external stimuli are exploited as solids. Figures 2A and S7 (in the SI) display the UV–visible steady-state diffuse reflectance (transformed to K-M function), emission, and excitation spectra of UiO-66-(OH)_2_ in powder form. The diffuse reflectance spectrum (represented as K-M) is similar to that observed in suspension and consists of a broad band with its intensity maximum at ∼385 nm (pale-yellow colored powder) and a hump at ∼350 nm (Figures S6B and S7). The emission spectrum is also comparable to that observed in suspension, showing two bands with maxima at ∼465 and ∼525 nm (Figures 2A and S6B). In accordance with our previous discussion and with other reports, these emission bands are attributed to the emission of the enol (∼465 nm) and keto (∼525 nm) tautomers of the DHT linker in the MOF.^27,33,37,53,54^ Moreover, the emission of the keto tautomer increases with higher excitation energies, as a consequence of a more favored ESIPT reaction, as explained in the previous section. The excitation spectrum of UiO-66-(OH)_2_ MOF in the solid state is characterized by a very broad band with multiple shoulders (Figure S7 in the SI). This might indicate the coexistence of different species, probably because of intramolecular hydrogen bonding interactions both in the ground and S_1_ states of the DHT linker. Interestingly, the emission spectrum of the pristine DHT linker in the solid state only shows the emission band of the enol tautomer (Figure S8 in the SI), indicating that the ESIPT reaction does not take place in the solid state of this molecule, and therefore, this process is favored by the coordination of the carboxylic acid to the Zr metal clusters.

The picosecond photodynamics of UiO-66-(OH)_2_ powder were explored by exciting the sample at 371 nm and recording the decays at different wavelengths (Figure 2B and Table 1). The emission decays of the MOF are much shorter than the ones observed for the linker in DMF and exhibit a different behavior depending on the interrogating region. In the bluest region (425–475 nm), corresponding to the emission of the enol tautomer, the decays were accurately fitted to a biexponential function giving time constants of τ_1_ = 45 ps and τ_2_ = 170 ps. On the other hand, in the reddest region (550–650 nm), corresponding to the emission of the keto tautomer, the analysis gives two time components of τ_1_ = 70 ps and τ_2_ = 272 ps. Hence, we can attribute the time constants obtained in the bluest region to the emission lifetime of different populations of DHT enol tautomers, while those found in the reddest region are the emission lifetimes of different populations of keto tautomers formed after the ESIPT reaction. Note that around 500 nm, both tautomers contribute to the emission decays, and the obtained time constants (52 and 216 ps) are a combination of the lifetimes of the enol and keto tautomers. Hence, our results from the photodynamics of UiO-66-(OH)_2_ powder, which have not been previously investigated in detail, enable shedding more light on the spectroscopic and photodynamical properties of this ESIPT-based MOF.

**Table 1:** Values of Time Constants (τi), Normalized to 100 Amplitudes (*a*_*i*_), and Contributions (*c*_*i*_) Obtained from the Analysis of the Emission Decays of UiO-66-(OH)_2_ in Powder Form upon Excitation at 371 nm and Observation As Indicated (Estimated Error Is around 10–15%)

sample	λ_obs_ (nm)	τ_1_/ps	*a*_1_	*c*_1_	τ_2_/ps	*a*_2_	*c*_2_
UiO-66-(OH)_2_ solid state	425	45	83	57	170	17	43
	435	45	86	62	170	14	38
	450	45	84	59	170	16	41
	475	45	81	53	170	19	47
	500	52	79	47	216	21	53
	550	70	69	36	272	31	64
	600	70	70	37	272	30	63
	650	70	67	34	272	33	66

### 3.5 Luminescent Detection of External Stimuli by UiO-66-(OH)_2_

In the following subsections, we will leverage the ESIPT reaction happening in the DHT linker of UiO-66-(OH)_2_ MOF to prove the ability of this material to detect multiple external stimuli (e.g., acid and base vapors and changes in the temperature and pressure). These results will demonstrate the potential of this material to be deployed in the development of advanced luminescent sensors and could be extrapolated to different luminescent ESIPT-based MOFs, expanding the knowledge in the field, and opening novel avenues for further designing and fabricating more efficient sensing materials.

#### 3.5.1 Detection of Acid and Base Vapors by a “Turn-On” Mechanism

As stated above, there is an urgent need for developing chemical sensors to guarantee a safety environment. Since many of the chemical pollutants produced in industry and other sectors are in the gas phase, it is required that the sensors must be capable of detecting those volatile products. Different techniques such as gas chromatography (GC), mass spectrometry (MS), flame ionization detection (FID), and optical absorption spectroscopy, among others, have been proposed.^55,56^ However, none of these reunites the desired properties (portability, economically viable, simple operativity, high sensitivity, and selectivity) of a chemical sensor all at once. As explained above, a promising alternative solution resides in the development of luminescent sensors that can be portable, low-cost, and easy-to-use, with a high selectivity and sensitivity. Among the possible luminescent sensors, those based on a “turn-on” (i.e., the detection of the analyte induces an emission intensity enhancement) and/or “wavelength-shifting” (i.e., the detection of the analyte produces a change in the emission color) are highly preferred, since some undesired effects (i.e., photodegradation) are minimized in comparison with the typical luminescent “turn-off” sensors. However, for a real boost in the field, it is necessary to design novel potential materials that can be employed in the fabrication of these types of sensors. Herein, we take advantage of the ESIPT reaction in UiO-66-(OH)_2_ MOF to detect the vapor of HCl and Et_3_N, which are toxic chemicals widely employed in different industrial processes. Although this MOF has been used for detecting anions and cations in solution,^27,35,36^ to the best of our knowledge, there are no examples of the detection of acid and base vapors. Similarly, most of the Zr-based MOFs reported up until now are used as sensors in solution.^11,12^ However, we have recently reported on the first example of a proton-transfer dye encapsulated within a MOF for the detection of acid and base vapors,^10^ so the present example is a further step to the use of ESIPT-based MOFs for this type of application.

**Figure 3:**
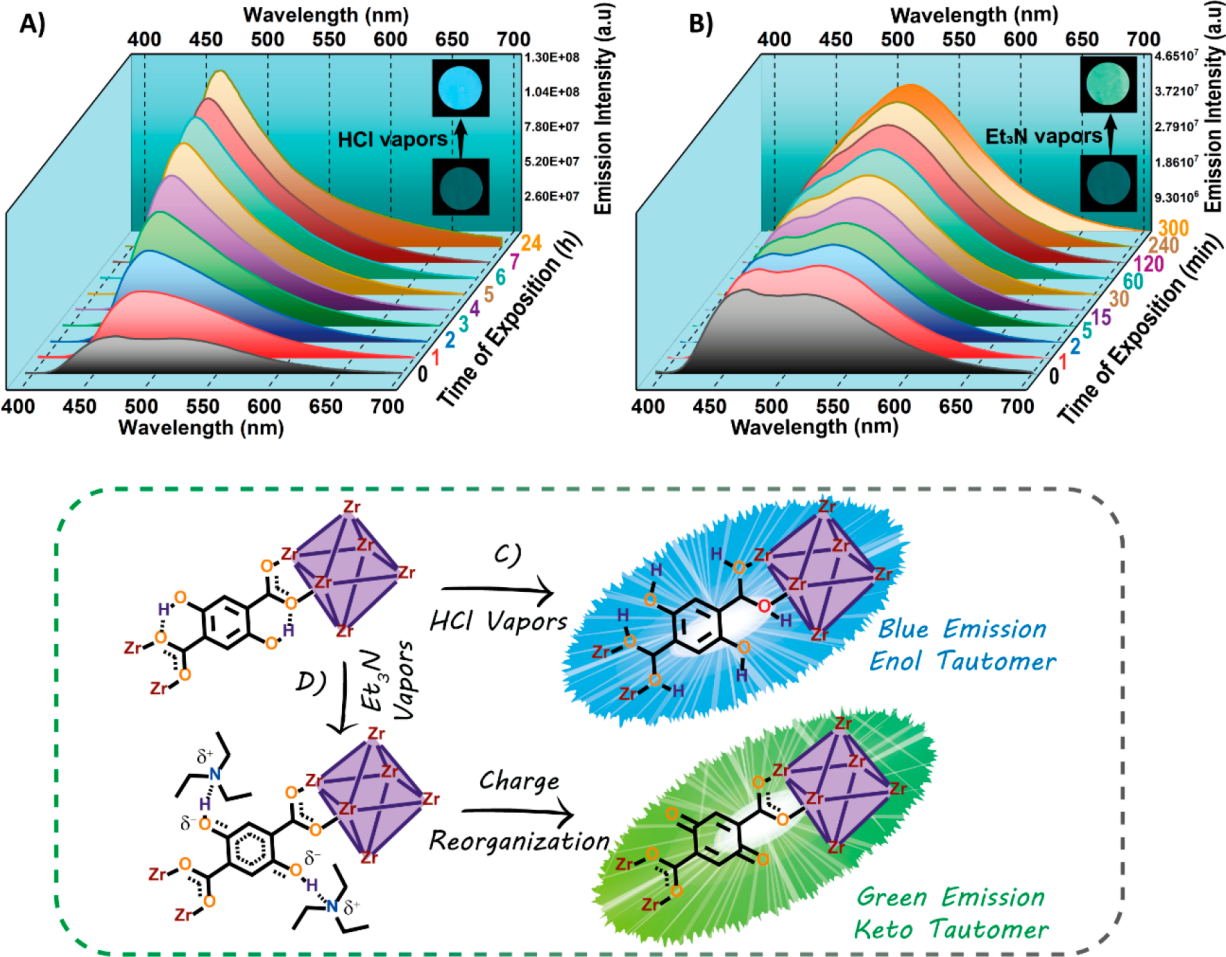
(A, B) Emission spectra of UiO-66-(OH)_2_ after different times of exposition to a saturated atmosphere of (A) HCl and (B) Et_3_N. The samples were excited at 380 nm. The inset photos show the real luminescence of the MOF material before and after its exposure to the corresponding vapors under irradiation with UV light (365 nm). (C, D) Schematic representation of UiO-66-(OH)_2_ in powder form after its interaction with a saturated atmosphere of (C) HCl and (D) Et_3_N vapors.

First, we have exposed the UiO-66-(OH)_2_ MOF powder to a saturated atmosphere of HCl, and its emission spectra were collected prior to and after its interaction with the analyte during different times of exposure (from 1 to 7 h, Figure 3A). Remarkably, the emission spectrum of UiO-66-(OH)_2_ MOF exhibits two noticeable changes upon interacting with HCl vapors: (i) a continuous increase in its emission intensity and (ii) the emission spectrum becomes a single band (maximum intensity at 465 nm), with the disappearance of the band at 525 nm observed for the pristine MOF (Figure 3A). These two observations reflect the potential of this MOF material to detect the vapor of HCl following turn-on and wavelength-shifting mechanisms (see the change in emission color in the photos of Figure 3A). The strong decrease or vanishing of the 525 nm emission band (keto tautomer emission) indicates that the ESIPT reaction is blocked when the MOF interacts with HCl. Based on this observation, we propose that the presence of HCl should protonate the carboxylate groups of the DHT linker, and consequently, the H atom of the −OH group cannot be transferred to those carboxylate functional groups (Figure 3C). Hence, the emission of the MOF is due to the enol tautomer of the DHT linker. Furthermore, the excitation spectrum of the UiO-66-(OH)_2_ MOF after its interaction with HCl exhibits a narrowing in comparison with the excitation spectrum of the pristine MOF (Figure S9A), suggesting a decrease in the possible intramolecular H-bond populations due to the protonation of the carboxylate groups in the linker (Figure 3C).

Since the used HCl (37%) contains water, we also explored the possible effect exercised by water molecules to the emission properties of the MOF. To this end, the material was exposed to a high humidity atmosphere (85%) under experimental conditions similar to those used for the detection of HCl vapors. As shown in Figure S10A, the emission spectra of the MOF exhibit a slight and progressive quenching with the exposition time to humidity, contrary to what we observed when interacting with HCl vapors (emission enhancement). There is a special difference in the emission band at 465 nm (enol species), which in the presence of HCl increases its intensity by almost 4 times, while in the presence of humidity, it slightly decreases (Figure S10B). Hence, this experiment further supports our proposed mechanism on the HCl detection and rules out a strong effect of water on the sensing of HCl vapors.

Moreover, to explore the stability of this MOF material in the presence of HCl vapor, we have measured the PXRD patterns and FTIR spectra of UiO-66-(OH)_2_ before and after its interaction with HCl for 7 h (Figure S11). The PXRD patterns show no remarkable changes, reflecting the structural robustness of this material (Figure S11A). On the other hand, the FTIR spectrum of the material before and after interacting with HCl remains almost the same, with one exception, which is a broadening of the band at 3400–2800 cm^–1^ (Figure S11B). This change further reinforces the proposed mechanism, where more protonated species are formed upon the interaction of UiO-66-(OH)_2_ with the HCl vapor. To rule out that the broadening of the band at 3400–2800 cm^–1^ was caused by the presence of water molecules, we have also measured the FTIR of the MOF after being exposed to a high humidity atmosphere for 7 h (Figure S11B), showing a different broadening (shifting to higher wavenumbers) than that observed when the UiO-66-(OH)_2_ interacts with the HCl, further evidencing our previous attribution.

We also explored the luminescence response of the UiO-66-(OH)_2_ MOF to the presence of vapors of Et_3_N, a largely used base compound. As shown in Figure 3B, the interaction with Et_3_N for just 1 min induces a significant change in the emission spectrum of UiO-66-(OH)_2_. In this case, we observed that prolonged exposure to Et_3_N vapor produces an increase in the emission intensity of the band at 525 nm. Hence, the luminescent response is based on a turn-on mechanism and a change in the emission color, since the ratio between the 465 and 525 nm bands is varying. These changes in the emission can be observed even by the naked eye (see the photos in Figure 3B). Moreover, the increase in the emission intensity is observable up to 4 h of interaction with vapors of Et_3_N, where a plateau is reached, and no more changes are detected. The excitation spectrum of UiO-66-(OH)_2_ after being exposed to Et_3_N is similar in shape to that recorded for the pristine MOF, except an increase in the intensity of the shoulder at ∼350 nm (Figure S9B).

The proposed mechanism for the detection of Et_3_N is opposite the one explained for the HCl. In this case, the Et_3_N vapors interact with the −OH groups of the DHT linker, triggering a deprotonation of these functional groups, and conferring the oxygen atoms with a negative charge (Figure 3D). This is followed by a charge reorganization, leading to the formation of the keto tautomer, whose emission maximum falls in the region of ∼525 nm (as previously demonstrated), reflecting the increase in intensity of this band. Similar mechanism has been proposed to explain the response of DHT-based MOFs to the presence of different chemicals in solution such as water or F^–^.^31,36,57^ The photoluminescence quantum yield of UiO-66-(OH)_2_ is weak, 0.34%, and it increases by ∼5 (1.60%) and ∼2 (0.55%) times in the presence of HCl and Et_3_N, respectively. This further corroborates the turn-on sensing mechanism. Moreover, we performed experiments to test the possible reversibility of the sensing behavior and observed that once the MOF interacts with the analytes, it does not recover its initial emission.

Finally, we have also characterized the structural and chemical properties of the UiO-66-(OH)_2_ after its exposition to Et_3_N vapors for 5 h. The PXRD data show no major differences with that obtained for the pristine MOF, corroborating the structural robustness of this MOF under the experimental conditions used (Figure S11A). On the other hand, the FTIR spectrum of the material before and after interacting Et_3_N vapors remains unaltered, with just one exception: a decrease in the intensity of the band at 3400–2800 cm^–1^ (Figure S11B). This decrease reflects a decrease in the population of the absorbing hydroxyl groups, in agreement with our proposed mechanism, where the interaction of Et_3_N with the MOF induces deprotonation of the linker.

#### 3.5.2 Mechanoluminescent Response of UiO-66-(OH)_2_

There is an increasing demand for developing sensors that are responsive to external physical stimuli such as mechanical compression. It has been reported that when a mechanical compression is exercised on the isostructural UiO-66 MOF, a bond breakage between the Zr atom and the carboxylate group of the organic linker occurs, leading to the formation of a free monodentate carboxylate group.^42^ Hence, we will leverage the ESIPT phenomenon alongside the mentioned bond-breakage to explore the potential applicability of UiO-66-(OH)_2_ to detect changes with the exercised pressure.

**Figure 4:**
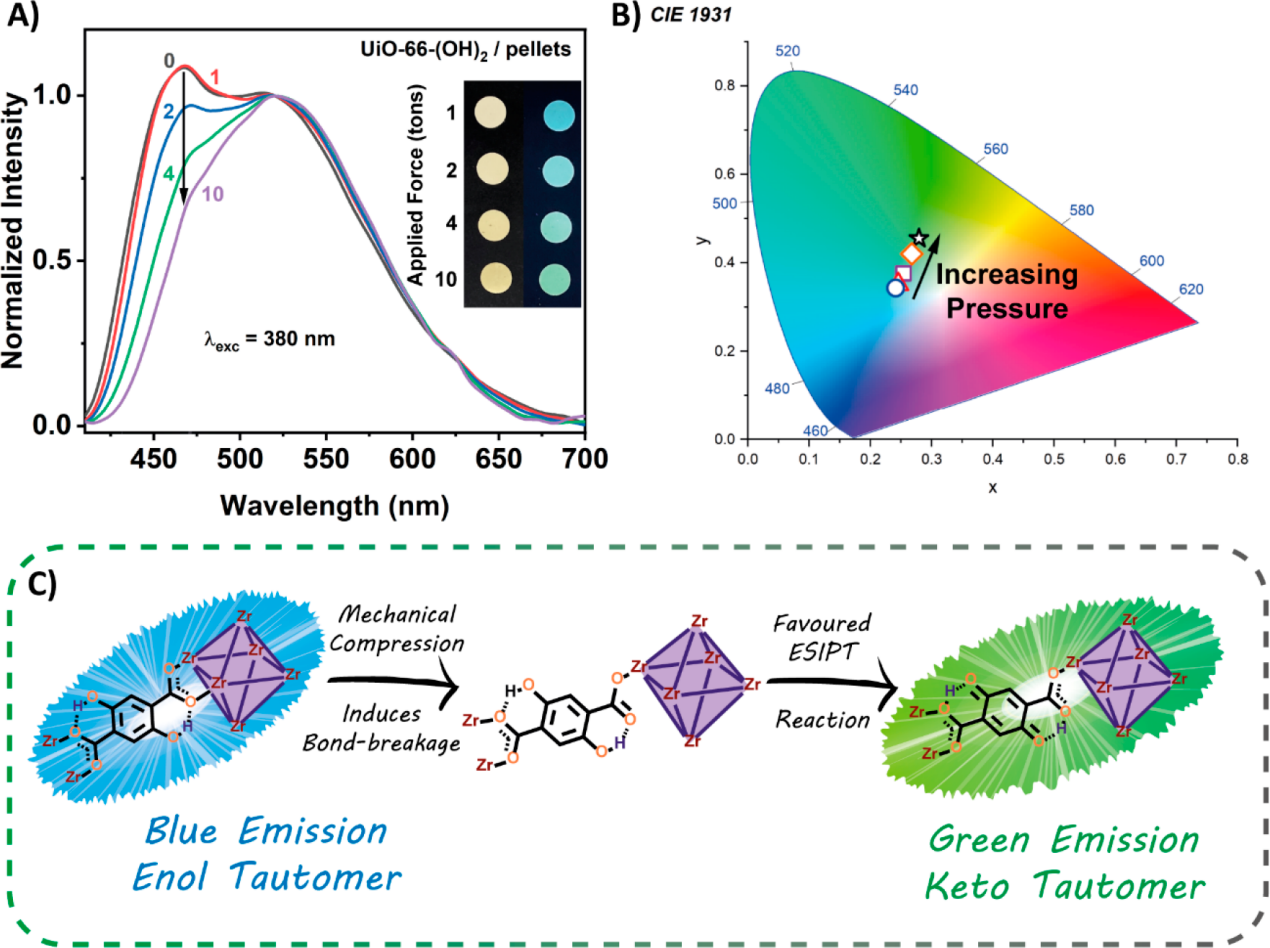
(A) Normalized (to the keto emission band, 525 nm) emission spectra of pellets compressed at different pressures (0, powder; 1, 1 ton; 2, 2 tons; 4, 4 tons; 10, 10 tons). The pictures are real photos of the UiO-66-(OH)_2_ pellets under daylight (left) and UV light (365 nm; right) irradiation. (B) Representation of the CIE coordinates of the pellets compressed at different pressures. (C) Proposed mechanism of mechanical compression detection by UiO-66-(OH)_2_ based on the ESIPT reaction.

To this end, we have prepared a total of four pellets (diameter of 1.3 cm), using 120 mg of UiO-66-(OH)_2_ for each pellet and applying different nominal stresses of 1, 2, 4, and 10 tons, respectively. Then, the fluorescence spectra of all these pellets were recorded and compared with that obtained for the MOF powder at atmospheric pressure. Figure 4 shows that the ratio between the intensity of the emission bands with a maximum at 465 nm (enol form) and 525 nm (keto tautomer) decreases with applied pressure. This change can even be detected by the naked eye. Upon irradiating the pellets with 365 nm UV light, the emission color changes from blue (1 ton) to green (10 tons, inset in Figure 4A) and matches well with the CIE coordinates as depicted in Figure 4B. These observations indicate the formation of a higher population of keto tautomers upon applying pressure.

The detection mechanism can be explained by considering the changes in the MOF structure (bond-breakage between the organic linker and the metal clusters) and the luminescent properties (ESIPT-based mechanism) of the organic linker. Upon applying increasing uniaxial mechanical compression to the fabricated pellets, the number of broken bonds between the Zr and oxygen of the COO group (Zr–O_COO_) increases. Indeed, it was estimated that nearly half of the bonds are fragmented after applying a pressure of ∼10 tons.^42^ Hence, the applied force induces the formation of more free uncoordinated C=O groups, which might act as proton acceptor agents, favoring the ESIPT reaction within the linker and therefore modulating the final color emission by the formation of a larger population of keto tautomers (Figure 4C). Moreover, the applied pressure can bring closer the functional groups involved in the ESIPT reaction, and consequently, there might exist H-bond interactions even in the ground state of the linkers. This possibility is further reinforced attending to the excitation spectra of the pellets. As shown in Figure S12A, a new band appears at ∼450 nm and its intensity grows with the applied pressure. This red-shifted band could be ascribed to the possible H-bonding interactions taking place in the ground state of the organic linkers. The emission spectra of the pellets exciting at 450 nm display their maximum at ∼525 nm (keto emission, Figure S12B), corroborating that the observed excitation band corresponds to keto tautomers. Notice that there exists the possibility that the MOF pellets might capture moisture from the ambient environment; however, under these experimental conditions, it did not affect their spectroscopic properties. In fact, the observed response is an increase in the emission intensity of the band at 525 nm, opposite what we observed when the MOF was subject to a higher humidity (85%) exposure for longer times (Figure S10). Moreover, the emission intensity of the pellet with just 1 ton remains the same as the initial spectrum of the powder MOF sample (Figure 4A), also suggesting that the possible presence of moisture under these experimental conditions does not really affect the spectroscopic properties.

We also measured the PXRD patterns of the pellets to confirm the proposed bond breakage mechanism. As displayed in Figure S13A, the peaks in the region between 10 and 35° begin to vanish with the applied pressure. Moreover, the two most intense peaks (at 7.5° and 8.6°) also experienced a decrease in their intensity alongside with a broadening. These observations reflect the loss of crystallinity of the MOF material with the exercised pressure, further corroborating our mechanism based on the bond-breakage between Zr and the oxygen of the COO group of the linker. This mechanism also explains why the emission of the MOF after being subjected to a pressure of 10 tons cannot be recovered to its initial value (Figure S13B). This is of special interest as this material could be exploited as a smart sensor for packaging, given that if an overpressure is exercised on that package, it will retain the information that can be later evaluated by fluorescence spectroscopy.

#### 3.5.3 Thermoluminescent Response of UiO-66-(OH)_2_

**Figure 5:**
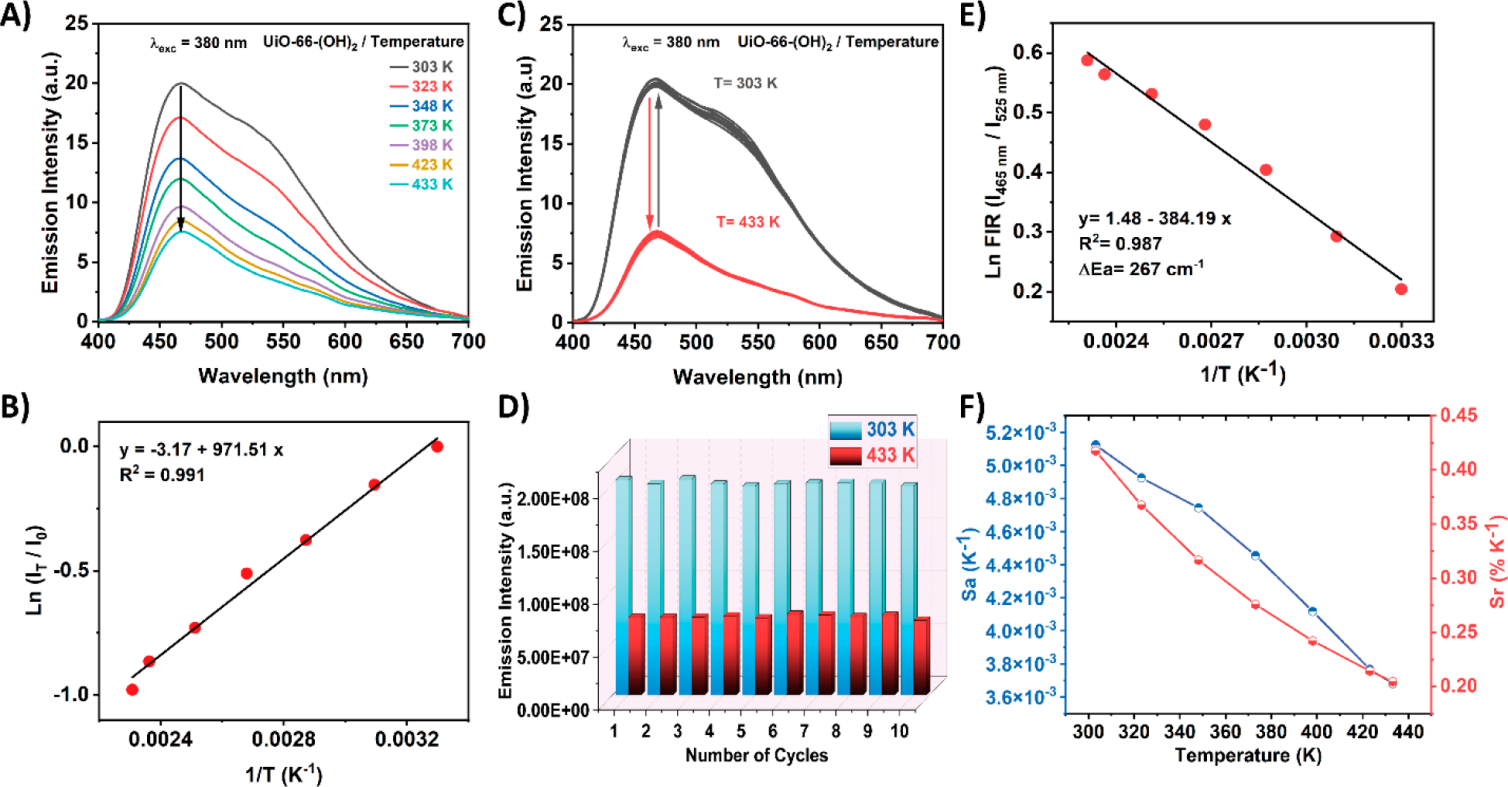
(A) Emission spectra of UiO-66-(OH)_2_ in powder form collected at different increasing temperatures. (B) Representation of the Arrhenius analysis, showing the linear response of the UiO-66-(OH)_2_ with the temperature. The values of *I*_T_ and *I*_0_ were recorded at 465 nm. (C) Emission spectra of UiO-66-(OH)_2_ measured upon 10 cycles of heating (433 K) and cooling (303 K) of the sample. (D) Representation of the emission intensity maxima (at 465 nm) measured during 10 cycles of heating (433 K) and cooling (303 K), reflecting a high reproducibility. (E) Representation of the Arrhenius analysis considering the fluorescence intensity ratio (FIR) of the emission bands at 465 and 525 nm. (F) Representation of the absolute (*S*_A_) and relative (*S*_R_) sensitivities versus temperature.

The design and synthesis of luminescent materials able to detect changes in the temperature of the medium is also one important target of the scientific community,^13,58,59^ since luminescent thermometers can replace conventional ones in a multitude of industrial processes where electrical/magnetic fields, different environmental conditions, and/or high temperatures preclude the use of these types of thermometers.^16,60^ In addition to that, the possibility of detecting changes of temperature in microenvironments (e.g., cells) is of great importance to unveil biological events.^61,62^ Hence, herein we have tested the ability of UiO-66-(OH)_2_ to detect changes in the temperature in a range between 30 and 160 °C (303–433 K) via luminescent response. To this end, we recorded the emission spectra of UiO-66-(OH)_2_ at different temperatures. As shown in Figure 5A, the emission intensity of UiO-66-(OH)_2_ is gradually quenched (turn off mechanism) with an increment in the temperature. Similar intensity quenching can be observed in the excitation spectra of the MOF upon increasing the temperature (Figure S14). The decrease in emission intensity with the temperature can be explained in terms of the opening of nonradiative deactivation channels due to the increment of vibrational motions of the organic linker.^13^ In fact, a previous report has demonstrated that for the terephthalate linker in UiO-66 MOF, flips around the *C*_*2*_ symmetry axis and librational motions exist.^63^ In that work, it was established that the temperature dependence of the flipping motion follows an Arrhenius dependence with an activation energy barrier of only 30 kJ/mol. Remarkably, the Arrhenius analysis of the luminescence intensity change of UiO-66-(OH)_2_ with respect to the temperature also reflects a linear response, yielding to a lower value of the energy activation to dark states, Ea = 8.1 kJ/mol (Figure 5B, and corresponding section 1.3 in the SI). This lower energy value can be associated with the presence of hydroxyl groups in the DHT linker or even with the presence of DMF molecules that might decrease the torsional barrier. Notice that H-bond aromatic molecules interacting with H-accepting ones exhibit faster nonradiative decays due to H-bonding interactions.^64,65^ The observed linear response is highly desired for the potential integration of this MOF material into a luminescent thermometer device.

Another important characteristic that any LMOF must fulfill to be implemented in a luminescent thermometer is high reproducibility. To investigate this, the material has been subjected to several cycles of heating (433 K) and cooling (303 K). Figure 5C shows how the emission intensity of UiO-66-(OH)_2_ is quenched after heating up to 433 K and recovered upon cooling down to 303 K. The values of the emission intensity maximum at these two temperatures over 10 cycles are depicted in Figure 5D. From these results, it is clear that the luminescent response of UiO-66-(OH)_2_ toward changes in the temperature is very reproducible, and therefore, this material is a very promising candidate to be used as an active layer for the fabrication of a luminescent thermometer. Moreover, and most importantly, since the emission of UiO-66(OH)_2_ arises from two different species, whose emission intensity changes in a different way with temperature, we can use this material for ratiometric luminescent thermometry. These types of thermometers are based on the fluorescence intensity ratio (FIR) between two emissive species, and they have received tremendous attention over the past years, as they are one of the most promising devices for replacing conventional thermometers.^66−68^ It is generally accepted that the energy gap (ΔEa) for thermally coupled levels (TCL) must be on the order of 200–2000 cm^–1^, since smaller values may lead to the overlap of the two emissions, while larger values may produce an insufficient number of electrons in higher energy states.^69^ The ΔEa can be calculated by applying the Arrhenius equation using the FIR values instead of the usual emission intensity, following the next equation:
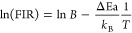
1

Hence, the value of ΔEa and *B* can be easily obtained from the slope (multiplied by *k*_B_, Boltzmann constant) and the intercept of the *y* axis, respectively. In our case, the calculated ΔEa is 267 cm^–1^ (Figure 5E), which is in the expected range of a TCL system. Other important parameters to consider and evaluate in an FIR thermometer are the relative and absolute sensitivities (*S*_A_ and *S*_R_, respectively), which are defined as^69^
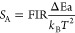
2
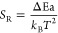
3

Figure 5F displays the values of *S*_A_ and *S*_R_ versus *T*, showing maximum values of *S*_A_ = 5 × 10^–3^ K^–1^ and *S*_R_ = 0.42 (% K^–1^). Even though these values are smaller than others reported for FIR materials,^66−68,70^ the main advantage is that our material is free of toxic and expensive rare-earth elements, and therefore, we consider that this type of MOF material can be a promising alternative for fabricating luminescent thermometers.

**Scheme 2:**
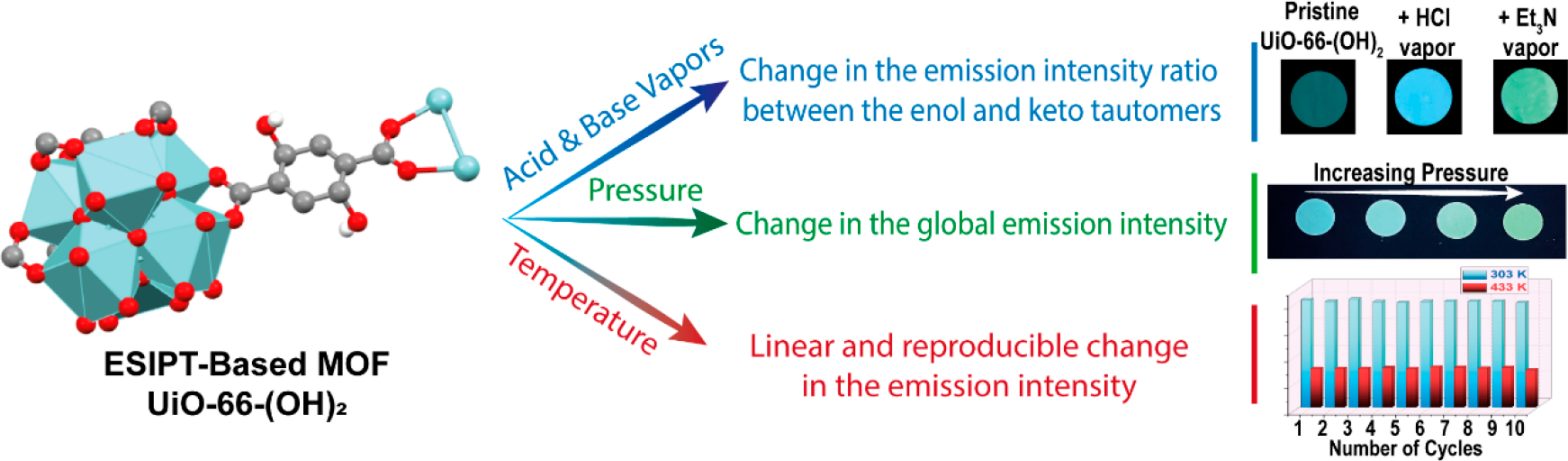
Illustration of the Observed Fluorescence Response of UiO-66-(OH)_2_ MOF to Chemical and Physical External Stimuli

To summarize, Scheme 2 illustrates our findings to use UiO-66-(OH)_2_ MOF as an advanced optical material for detecting chemical (e.g., base and acid vapors) as well as physical (e.g., changes in the applied pressure and temperature) external stimuli.

## 4 Conclusion

Herein, we have demonstrated for the first time how the UiO-66-(OH)_2_ MOF is a promising candidate to be employed as an active optical material for developing luminescent sensors of different external stimuli. To this end, we first examined the spectroscopic and photodynamics properties of the DHT molecule in different solutions, which were governed by the ratio of enol/keto population and, therefore, by the ESIPT reaction efficiency. Subsequently, a detailed study of the spectroscopic and photodynamic properties of UiO-66-(OH)_2_ in suspension and solid-state was carried out and compared to the results obtained for the pristine DHT linker. From these results, we unveiled that the emission of the MOF is mainly caused by the organic linker, and therefore, the emission of the material also depends on the ESIPT efficiency happening in the DHT linker (i.e., enol/keto ratio). Afterward, we assessed the potential of this material to detect different external stimuli, such as the presence of base and acid vapors or changes in temperature or applied pressure. The material was first exposed to saturated atmospheres of HCl and Et_3_N. When the MOF interacts with HCl vapors, there is an increase in the emission intensity of the band with a maximum at 465 nm (corresponding to the enol emission), reaching a plateau after 6 h of exposition. On the other hand, when the material was exposed to a saturated atmosphere of Et_3_N, we observe an almost instantaneous increase in the intensity of the 525 nm band (corresponding to the emission of the keto form), reaching a plateau after 4 h. A further remarkable finding is that when the material was subjected to increasing uniaxial compression, the ratio of the emission intensity between the bands at 465 and 525 nm decreases due to the increase of the keto tautomeric population. Last but not least, we also demonstrated that the UiO-66-(OH)_2_ MOF exhibits a linear and reproducible luminescent quenching response toward increments in the temperature from 303 to 433 K, as a consequence of the increase in the nonradiative deactivation channels. These results prove the potential of ESIPT-based LMOFs to be implemented in the development of advanced luminescent sensors of acid and base vapors, temperature, and mechanical compression and shed light for further design of more efficient ESIPT-MOF materials.

## Supporting Information Available

Results and discussion of the UV–vis steady-state absorption and emission spectra and time-resolved emission decays of the DHT linker in DMF and DMF containing HCl and Et_3_N. Other additional experimental details and data include PXRD pattern, FTIR spectrum, SEM images and N_2_ sorption isotherm of UiO-66-(OH)_2_, UV–vis steady-state data of DHT and UiO-66-(OH)_2_ in DMF and solid state, UV–vis steady-state spectra of UiO-66-(OH)_2_ after its interaction with the HCl and Et_3_N vapors and under 85% humidity, PXRD patterns and FTIR spectra of the UiO-66-(OH)_2_ MOF after its exposition to HCl and Et_3_N vapors and under 85% humidity, UV–vis steady-state and PXRD of UiO-66-(OH)_2_ pellets after compressed at different pressures, UV–vis steady-state excitation spectra of UiO-66-(OH)_2_ at different temperatures, and the Arrhenius analysis section (PDF)
