# Supplementary material for: Taking Advantage of a Luminescent ESIPT-Based Zr-MOF for Fluorochromic Detection of Multiple External Stimuli: Acid and Base Vapors, Mechanical Compression, and Temperature
Source: ACS Appl Mater Interfaces. 2023 Nov 20;15(48):56587–99. doi: 10.1021/acsami.3c14348 (PMC10711708; doi:10.1021/acsami.3c14348)
Supplement: Supplementary file 1 — am3c14348_si_001.pdf [file am3c14348_si_001.pdf]

Supporting Information

Taking Advantage of a Luminescent ESIPT-based Zr-MOF for Fluorochromic Detection of Multiple External Stimuli: Acid & Base Vapors, Mechanical Compression, and Temperature

Francisco Sánchez, Mario Gutiérrez, Abderrazzak Douhal**

Departamento de Química Física, Facultad de Ciencias Ambientales y Bioquímica, INAMOL, Universidad de Castilla-La Mancha, Avenida Carlos III, S/N, 45071 Toledo, Spain.

***Corresponding authors**

E-mail: Abderrazzak.douhal@uclm.es

E-mail: Mario.gutierrez@uclm.es

Index

Section 1		*Page 4*
Section 1.1: UV-Vis Steady-State Photophysical Properties of DHT linker in DMF solution		*Page 4*
UV-vis steady-state spectroscopy data of DHT in DMF	***Figure S1***	*Page 7*
Section 1.2: Time-Resolved Photodynamical Properties of DHT linker in DMF solution		*Page 7*
Magic-angle emission decays of DHT in DMF, DMF/HCl and DMF/Et3N.	***Figure S2***	*Page 8*
Multiexponential analysis of the emission decays of DHT in DMF, DMF/HCl, and DMF/Et3N	***Table S1***	*Page 8*
PXRD patterns and FTIR spectrum of UiO-66-$(OH)_2$	***Figure S3***	*Page 10*
SEM images of UiO-66-$(OH)_2$	***Figure S4***	*Page 10*
N_2 sorption isotherm of UiO-66-$(OH)_2$	***Figure S5***	Page 10
UV-vis steady-state spectroscopy data of DHT and UiO-66-$(OH)_2$ in DMF and solid state	***Figure S6***	*Page 11*
Normalized diffuse reflectance and excitation spectra of UiO-66-$(OH)_2$ in solid state	***Figure S7***	*Page 11*
Normalized diffuse reflectance and emission spectra of DHT in solid state	***Figure S8***	*Page 12*
Normalized excitation spectra of UiO-66-$(OH)_2$ in solid state after its exposure to a saturated atmosphere of HCl and Et_3N vapors	***Figure S9***	*Page 12*
Emission spectra of UiO-66-$(OH)_2$ after different times of exposition to an atmosphere of 85% of humidity and comparison of the emission intensity of UiO-66-$(OH)_2$ after its exposure to a saturated atmosphere of HCl and to an atmosphere with an 85% of humidity	***Figure S10***	*Page 13*

PXRD patterns and FTIR spectra of UiO-66-$(OH)_2$ after its interaction with HCl, Et_3N and to an atmosphere with an 85% of humidity	***Figure S11***	*Page 13*
Normalized emission spectra of UiO-66-$(OH)_2$ after compressed at different applied pressures	***Figure S12***	*Page 14*
PXRD patterns of the UiO-66-$(OH)_2$ pellets after being compressed at different pressures and emission spectra of the MOF before and after being pelletized at 10 tons, and after being grounded with a pestle and a mortar	***Figure S13***	*Page 14*
Normalized excitation spectra of UiO-66-$(OH)_2$ in solid state at different temperatures	***Figure S14***	*Page 15*
Section 1.3: Arrhenius Analysis		*Page 15*
References		*Page 16*

Section 1

Section 1.1: UV-Vis Steady-State Photophysical Properties of DHT linker in DMF solution

Prior investigating the luminescent properties of UiO-66(OH)$_2$, we have studied the steady-state UV-Vis spectroscopic behavior of the 2,5-dihydroxyterephthalic acid (DHT) linker in DMF solution (10^{-5} M), and in presence of HCl and Et_3N. **Figure S1** displays the corresponding absorption and emission spectra. The absorption spectrum of DHT in DMF is a single band with its maximum centered at 370 nm, while its emission spectrum is a broad band (FWHM = 3230 cm^{-1}) with its maximum located at ~525 nm (**Figure S1A**). The large Stokes shift ($\Delta\nu_{Stokes}$ = 7980 cm^{-1}) indicates the occurrence of an ESIPT process, and thus, the emission can be attributed to the keto tautomer. Similar emission band has been previously observed for this linker and was attributed to the emission of a keto structure formed through an ESIPT reaction in the excited enol species.[1, 2] However, as the linker contains two −OH functional groups, we suggest that two different keto tautomers might be formed upon excitation of the enols to the S1 state, mono− and di−keto tautomers (see **Scheme 1**).[3, 4]

To shed more light on the spectroscopic properties of the possible conformers of DHT linker, we added 0.972 g of HCl (37%) to the DMF solution to stabilize the enol species. The absorption spectrum of DHT in the DMF/HCl solution is comparable to that found in pure DMF, however, the emission spectrum is different and consists of a combination of 2 bands (**Figure S1B**). The red-shifted band is similar to that observed in pure DMF, with its maximum at ~525 nm, and therefore, can be assigned to the emission of the keto tautomers. However, the new blue emission band has its intensity maximum located around 450 nm (**Figure S1B**), being the Stokes shift just 4805 cm^{-1}. Thus, this band can be ascribed to the emission of the enol tautomers, as previously described for other DHT-

based MOFs.[1, 5-7] The presence of HCl might partially protonate the C=O groups of the carboxylic acids of DHT molecules, hindering the ESIPT reaction, and stabilizing the enol tautomers.

The steady-state spectroscopic properties of DHT in DMF solution and in presence of 0.602 g of Et_3N were also explored to get information on the possible existence of anionic species. As shown in **Figure S1C**, the absorption spectrum of DHT in DMF/Et_3N is blue shifted (absorption maximum at 355 nm) while the emission spectrum is red shifted (emission maximum at 570 nm) when compared to the spectra obtained in pure DMF (370 and 525 nm, respectively). It is well-known that ESIPT molecules might present H-bond interactions in their ground-state which stabilize the structure, shifting the absorption maximum towards longer wavelengths.[8-10] Hence, in presence of Et_3N, the −OH groups of DHT linker can interact with the Et_3N molecules, breaking the pre-existent intramolecular H-bond interactions in DHT molecule, and inducing a blue shift in the absorption maximum. Upon photoexcitation, the DHT molecule will transfer the H atoms to the Et_3N molecules (excited state intermolecular proton transfer, ESPT) leading to the formation of an anionic specie, which is emitting at wavelengths (570 nm, **Figure S1C**) longer than the keto tautomer (525 nm, **Figure S1A**). Moreover, the FWHM of this band is also very broad, with a value of ~ 4328 cm^{-1}, suggesting the emission of different anionic species, as there are many functional groups that can be deprotonated (**Scheme 1**). However, we cannot fully discard that even in presence of Et_3N, some DHT molecules might undergo an ESIPT reaction leading to the formation of keto tautomers.

Finally, the excitation spectra of DHT in the different environments are very similar to the absorption ones independently on the observation wavelengths (**Figure S1**), indicating a common origin of the excited species.

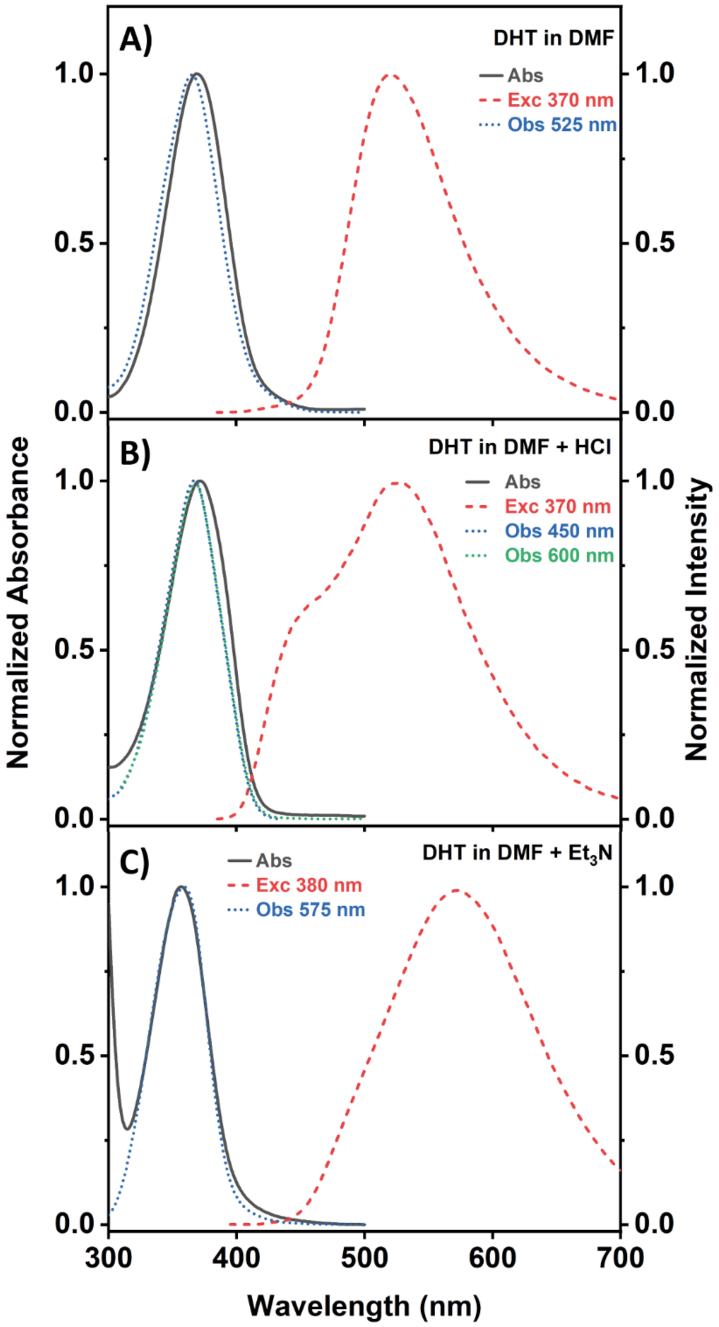

Figure S1. Normalized absorption (black solid line), excitation (dotted lines) and emission spectra (dashed lines) of DHT linker in **A)** DMF, **B)** DMF/HCl and **C)** DMF/Et_3N solutions. The different excitation and observation wavelengths are indicated in the figure.

Section 1.2: Time-Resolved Photodynamical Properties of DHT linker in DMF solution

The photodynamics properties of DHT in different media (DMF, DMF/HCl and DMF/Et_3N) were also investigated. **Figure S2** displays the corresponding emission decays upon excitation at 371 nm and recorded the signal at different wavelengths. **Table S1** shows the values of the time constants, amplitudes, and contributions obtained from the multiexponential global fit analysis of the decay traces. Firstly, we focus on the results obtained for DHT in DMF (**Figure S2A**). The emission decays of DHT in DMF were accurately fitted using a biexponential function, giving two time constants of τ_1= 2.6 ns and τ_2 = 8.6 ns. As explained above, the broad emission spectrum observed for DHT in DMF indicates the presence of different species at the first electronically excited state (S1) that can be ascribed to mono− and di−keto tautomers, and therefore, we suggest that the observed time components correspond to the emission lifetimes of these species. We propose that the longest component (τ_2) would correspond to the emission lifetime of the di-keto tautomer, since this structure will be more stabilized, while τ_1 would be the emission lifetime of the mono-keto tautomer (**Scheme 1**).

The photodynamics of DHT in presence of HCl (DMF/HCl mixture) is rather different, showing a dependency with the observation wavelength (**Figure S2B** and **Table S1**). In the bluest region (425 to 475 nm), the decays present a biexponential behavior with time constants of τ_1 = 1.1 ns and τ_2 = 4.3 ns, however, at lower energies (500 – 650 nm) the shorter time component (τ_1) vanishes. Since the emission of the enol tautomer appears at higher energies, we attribute the shorter component (τ_1), which contributes more to the signal in the bluest spectral region, to the emission lifetime of the enol species, while the longest one (τ_2) is the emission lifetime of the keto one. Note that the value of τ_2 is a mean value of the two lifetimes observed for DHT in pure DMF.

Finally, the photodynamics of DHT in presence of Et3N (DMF/ Et_3N mixtures) exhibits a much more complex behavior, giving three-time constants of τ_1= 411 ps, τ_2 = 3.1 ns, and τ_3= 8.3 ns (**Figure S2C** and **Table S1**). As explained above, the broad emission spectrum of DHT in DMF/Et_3N reflects the coexistence of different anionic and keto tautomers at the S1 state. Since τ_2 and τ_3 values are very similar to those found for DHT in pure DMF, we attribute them to the emission lifetime of the mono- and di-keto tautomers, while τ_1 (not observed in pure DMF) could be the emission of an anionic specie. However, we cannot rule out that other anionic structures might have similar emission lifetimes to that of the keto tautomers, and therefore, τ_2 and τ_3 components could be a result of the combination of the emission lifetimes of keto and anionic species.

Once the spectroscopic and photodynamical properties of the DHT linker were unraveled, we will now focus on the UiO-66-$(OH)_2$ structural, chemical, spectroscopic, and photodynamical properties.

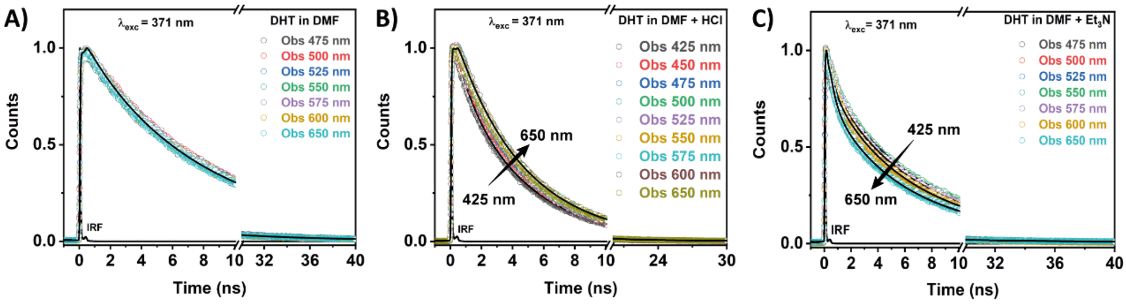

Figure S2. Magic-angle emission decays of DHT in **A)** DMF, **B)** DMF/HCl and **C)** DMF/Et_3N. The sample was excited with a 371 nm pulsed laser and probed at the indicated wavelengths. The solid black lines correspond to the best fit of the decays using a multiexponential function, while the IRF is the instrumental response function.

Table S1. Values of time constants (τ_i), normalized to 100 amplitudes (a_i) and contributions (c_i) obtained from the multiexponential analysis of the emission decays of DHT in DMF, DMF/HCl, and DMF/Et_3N upon photoexcitation at 371 nm. The estimated error is around 10 – 15%.

Sample	λ_{obs} (nm)	τ_1 / ns	a_1	c_1	τ_2 / ns	a_2	c_2	τ_3 / ns	a_3	c_3
DHT in DMF	**475**	2.6	10	3	8.6	90	97	-		
	**500**		4	1		96	99			
	**525**		5	2		95	98			
	**550**		6	2		94	98			
	**575**		8	3		92	97			
	**600**		9	3		91	97			
	**650**		11	4		89	96			
DHT in DMF + HCl	**425**	1.1	18	5	4.3	82	95	-		
	**450**		12	5		88	95			
	**475**		5	4		95	96			
	**500**	-	-	-		100	100			
	**525**					100	100			
	**550**					100	100			
	**575**					100	100			
	**600**					100	100			
	**650**					100	100			
DHT in DMF + Et_3N	**475**	0.41	25	2	3.1	23	13	8.3	52	85
	**500**		29	2		20	12		51	86
	**525**		26	2		17	9		57	89
	**550**		24	2		16	9		60	89
	**575**		25	2		19	11		56	87
	**600**		28	2		21	13		51	85
	**650**		34	3		24	17		42	80

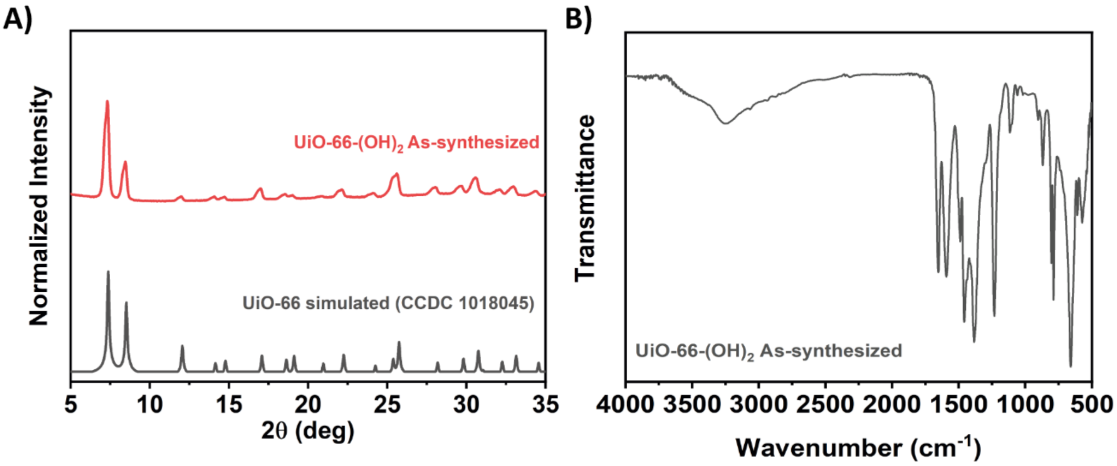

Figure S3. A) PXRD pattern and **B)** FTIR spectrum of UiO-66-$(OH)_2$. The graph **A)** includes the simulated PXRD pattern of UiO-66.

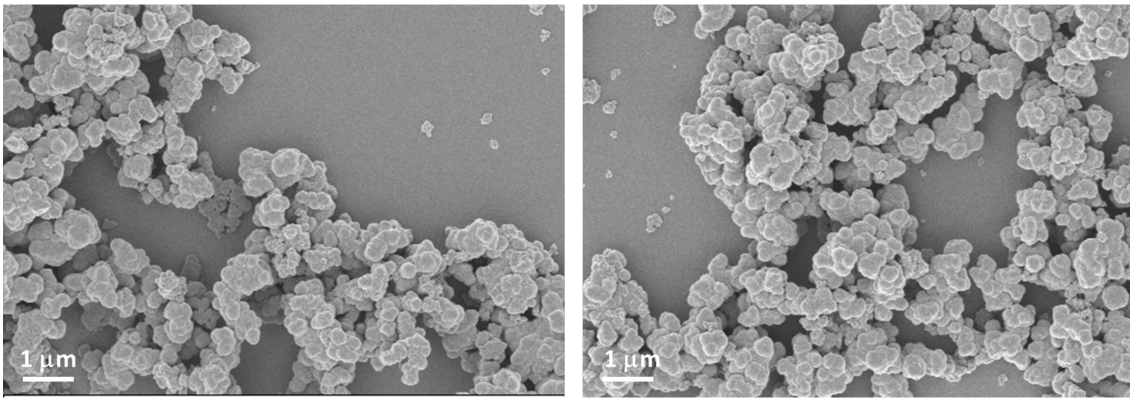

Figure S4. SEM images of UiO-66-$(OH)_2$.

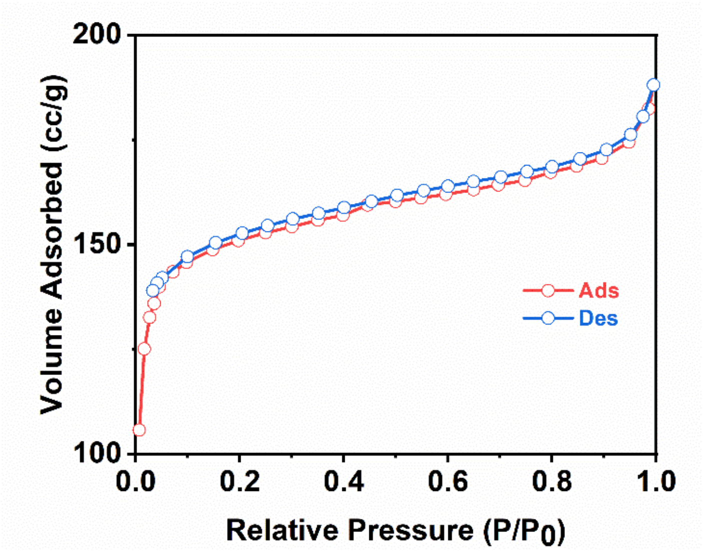

Figure S5. N_2 sorption isotherm of UiO-66-$(OH)_2$ at 77 K. The BET surface area of UiO-66-$(OH)_2$ is 560 $m^2\ g^{-1}$.

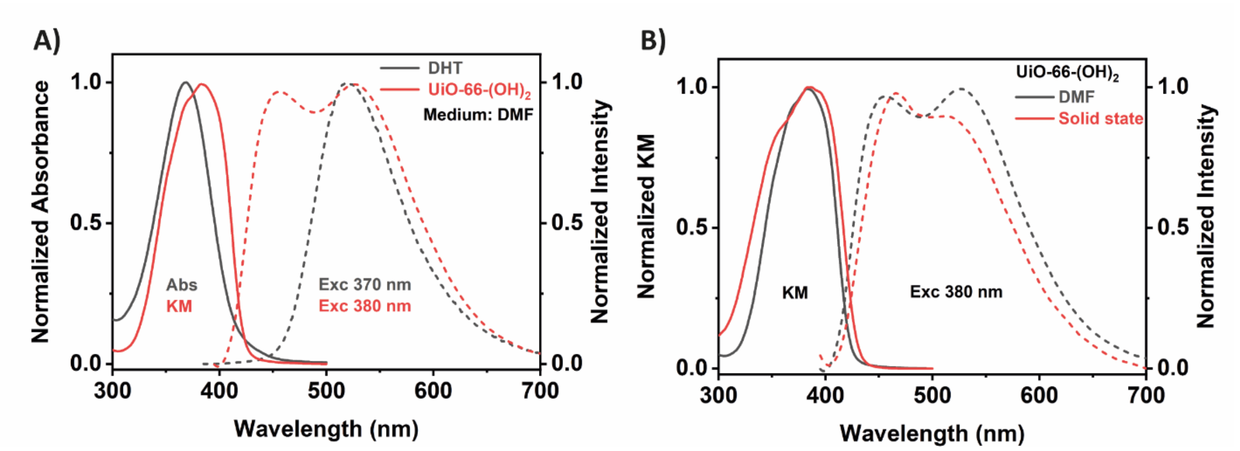

Figure S6. Normalized absorption, diffuse reflectance (converted to K–M function, solid lines) and emission spectra (dashed lines) of **A)** DHT and UiO-66-$(OH)_2$ in DMF and **B)** UiO-66-$(OH)_2$ in DMF and solid state. The excitation wavelengths are indicated in the inset.

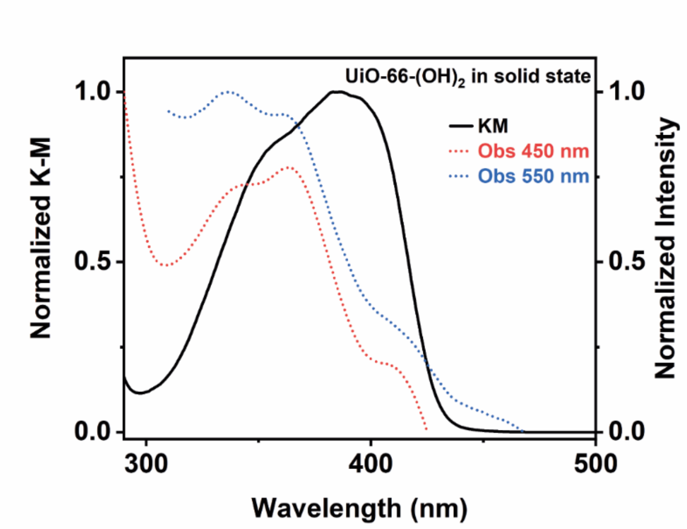

Figure S7. Normalized diffuse reflectance (converted to K–M function, black solid line) and excitation spectra (dotted lines) of UiO-66-$(OH)_2$ in powder form.

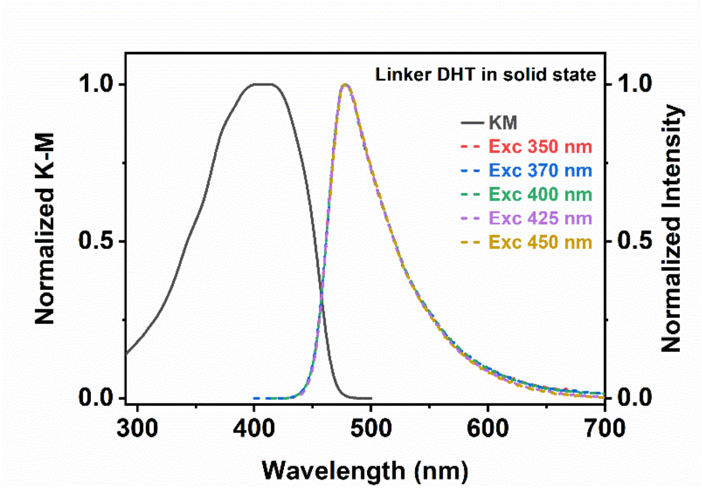

Figure S8. Normalized diffuse reflectance (converted to K–M function, black solid line) and emission spectra (dashed lines) of DHT in powder form. The excitation wavelengths are shown in the figure.

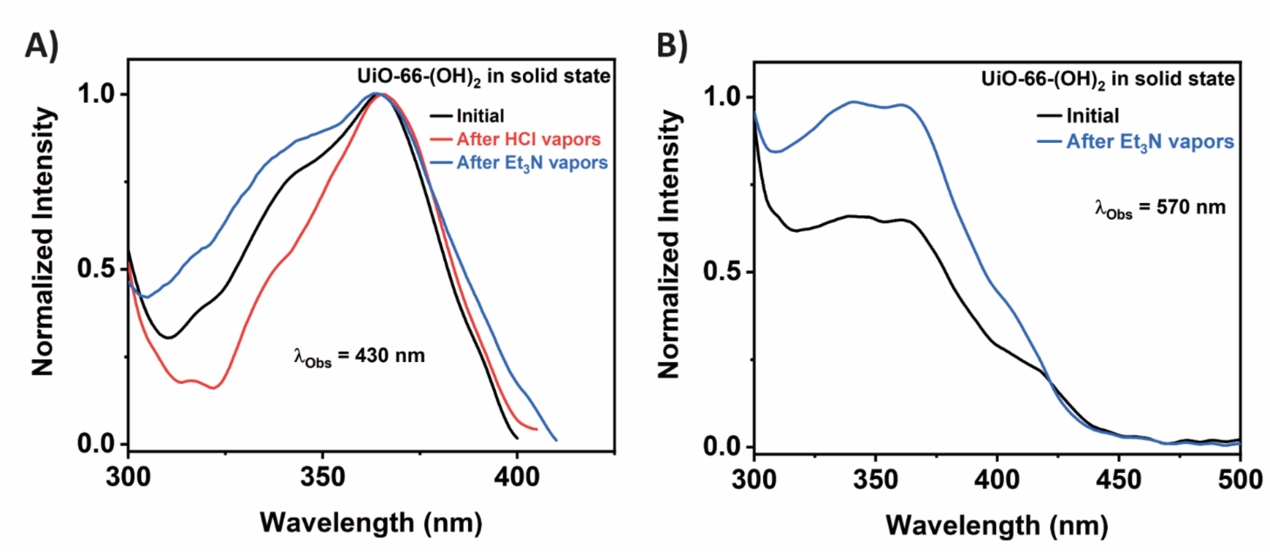

Figure S9. Normalized excitation spectra of UiO-66-$(OH)_2$ in powder form after its exposure to a saturated atmosphere of HCl and Et_3N vapors. **A)** Observation at 430 nm and **B)** at 570 nm.

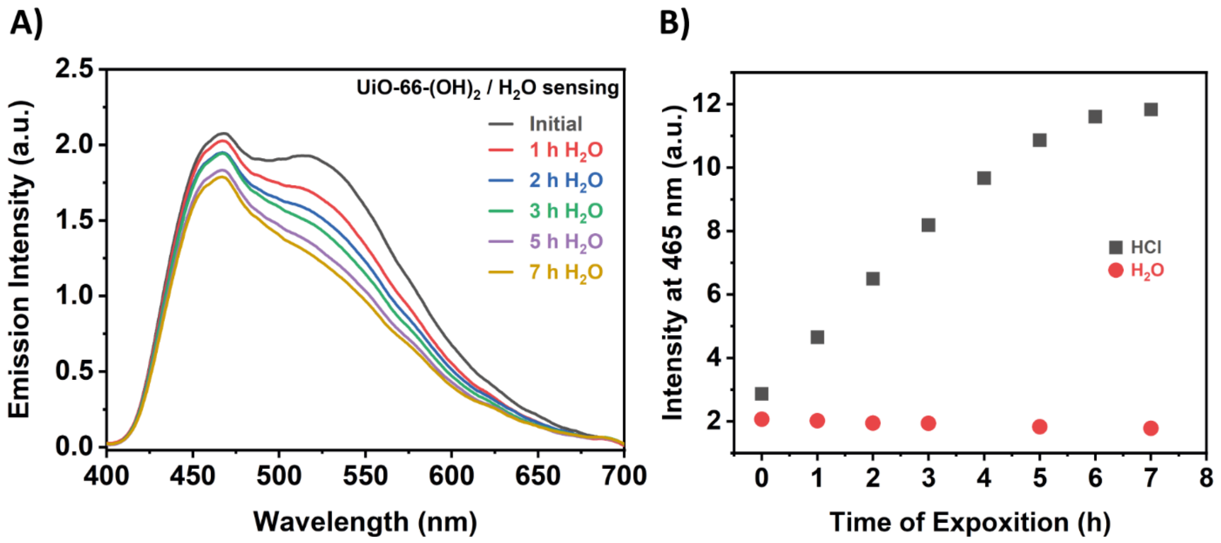

Figure S10. A) Emission spectra of UiO-66-$(OH)_2$ after different times of exposition to an atmosphere of 85% of humidity. The sample was excited at 380 nm. **B)** Comparison of the emission intensity (recorded at 465 nm) of UiO-66-$(OH)_2$ after its exposure to a saturated atmosphere of HCl (black squares), and to an atmosphere with an 85% of humidity (red dots).

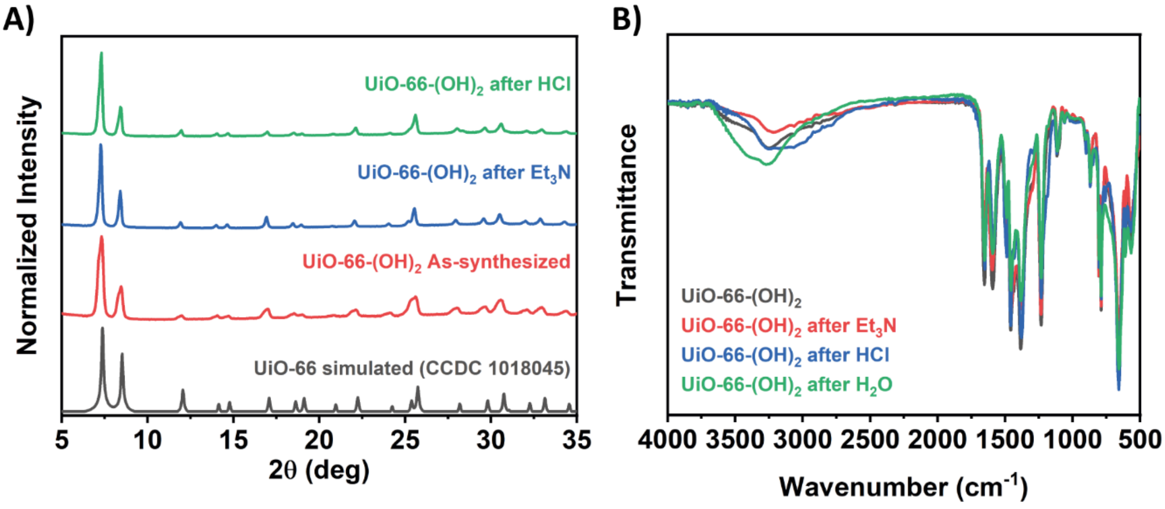

Figure S11. A) PXRD patterns and **B)** FTIR spectra of UiO-66-$(OH)_2$ before and after its exposure to saturated atmospheres of HCl (7 h), Et_3N (5 h), and to an atmosphere with an 85% of humidity (7 h) respectively. The graph in A) includes the simulated PXRD pattern of UiO-66 (CCDC 837796).

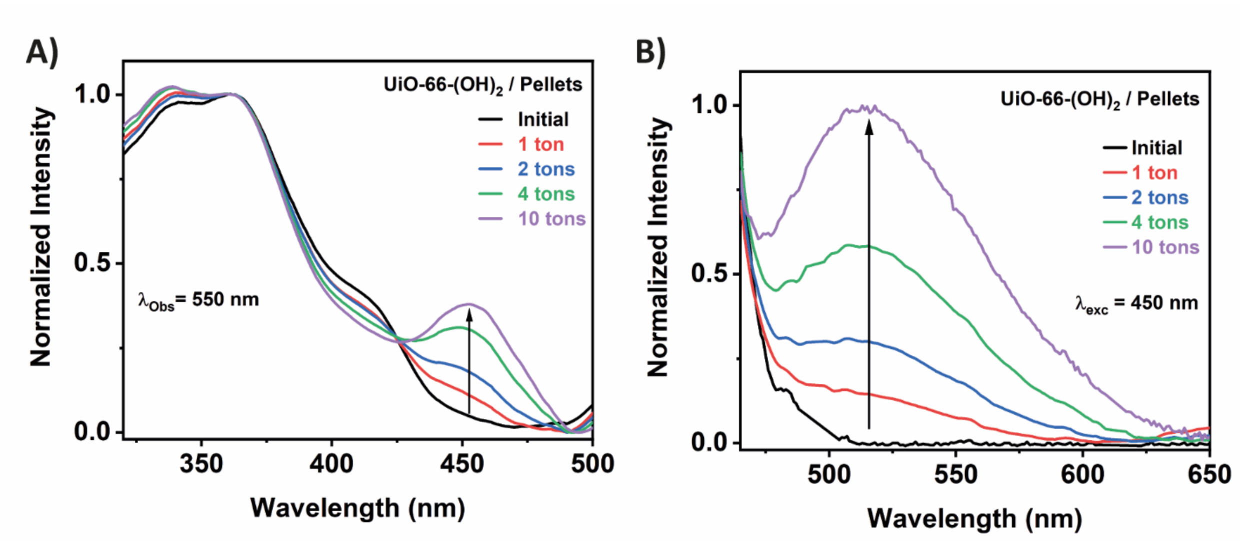

Figure S12. Normalized **A)** excitation and **B)** emission spectra of UiO-66-$(OH)_2$ pellets compressed at different pressures (indicated as inset). The excitation and observation wavelengths are indicated in the graphs.

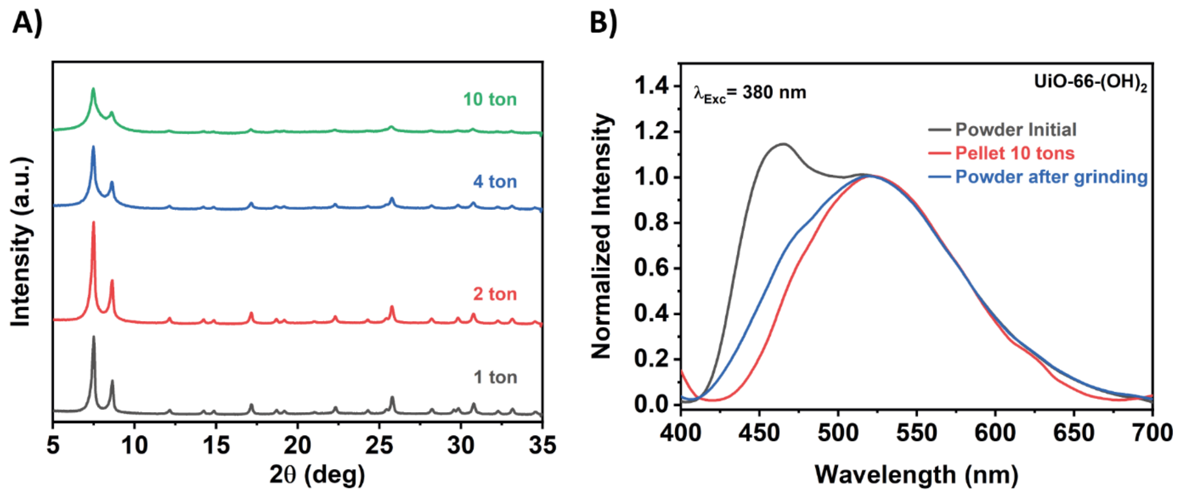

Figure S13. A) PXRD patterns of UiO-66-$(OH)_2$ pellets after being compressed at different pressures (1 ton, 2 tons, 4 tons, 10 tons), respectively. **B)** Normalized to 1 (at 525 nm) emission spectra of UiO-66-$(OH)_2$ in powder form (black line), in the form of a pellet after bein applied 10 tons of pressure (red line) and in powder form after grind the pellet (blue line). The samples were excited at 380 nm.

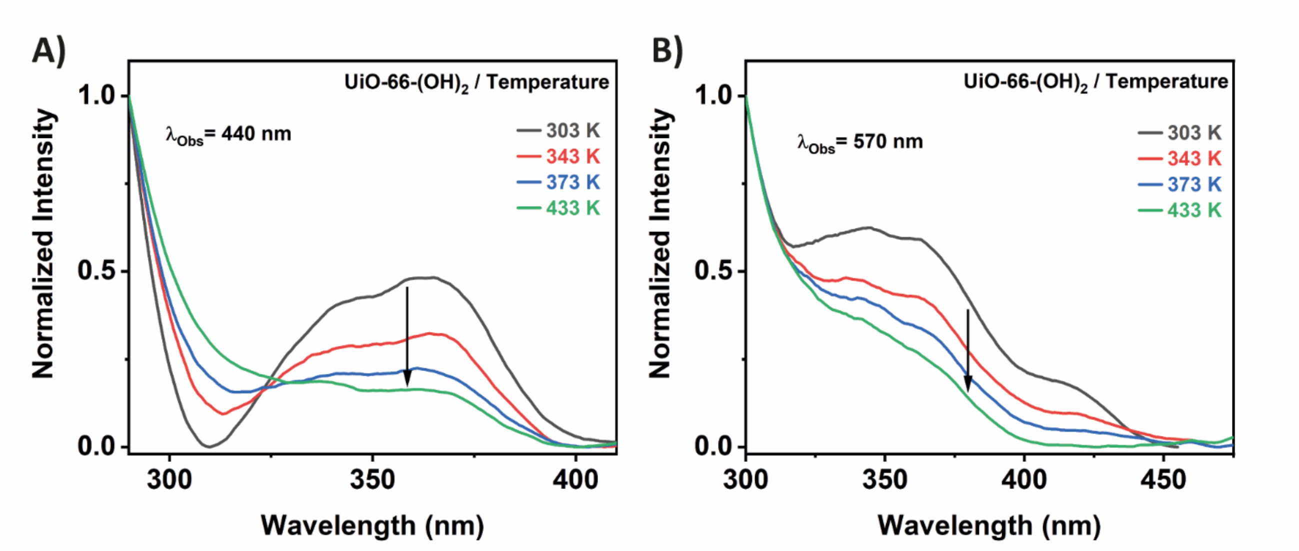

Figure S14. Normalized excitation spectra of UiO-66-$(OH)_2$ in powder form at different temperatures (indicated in the inset) recording the signal at **A)** 440 and **B)** 570 nm.

Section 1.3: Arrhenius Analysis

The activation energy barrier for the non-radiative processes happening in UiO-66-$(OH)_2$ MOF was calculated by applying the Arrhenius equation:

$$I(T) = \frac{I_0}{1 + Aexp\left(-\frac{\Delta E_a}{k_B T}\right)}$$

where I(T) and I_0 are the fluorescence intensities at T_n and T_0 (303 K), respectively, A is the preexponential Arrhenius factor, k_B is the Boltzmann constant and ΔEa is the activation energy barrier of non-radiative processes.

ACKNOWLEDGMENT

This research was supported by: PID2020-116519RB-I00 and TED2021-131650B-I00 funded by MCIN/AEI/10.13039/501100011033 and by the European Union (EU); SBPLY/19/180501/000212 and SBPLY/21/180501/000108 funded by JCCM and by the EU through "Fondo Europeo de Desarollo Regional" (FEDER); and 2020-GRIN-28929 funded by UCLM (FEDER). M.G. thanks the EU for financial support through Fondo Social Europeo Plus (FSE+). F. S. thanks Ministerio de Universidades for the FPU21/04332 national fellowship.

References

1. Bhattacharya, B.; Halder, A.; Paul, L.; Chakrabarti, S.; Ghoshal, D., Eye-Catching Dual-Fluorescent Dynamic Metal–Organic Framework Senses Traces of Water: Experimental Findings and Theoretical Correlation. *Chem. Eur. J.* **2016,** *22* (42), 14998-15005.
2. Huang, P.; Liu, Y.; Karmakar, A.; Yang, Q.; Li, J.; Wu, F.-Y.; Deng, K.-Y., Tuning the excited-state intramolecular proton transfer (ESIPT)-based luminescence of metal–organic frameworks by metal nodes toward versatile photoluminescent applications. *Dalton Transactions* **2021,** *50* (20), 6901-6912.
3. Serdiuk, I. E.; Roshal, A. D., Exploring double proton transfer: A review on photochemical features of compounds with two proton-transfer sites. *Dyes and Pigments* **2017,** *138*, 223-244.
4. Zhao, J.; Chen, J.; Liu, J.; Hoffmann, M. R., Competitive excited-state single or double proton transfer mechanisms for bis-2,5-(2-benzoxazolyl)-hydroquinone and its derivatives. *Phys. Chem. Chem. Phys.* **2015,** *17* (18), 11990-11999.
5. Lei, J.; Wang, B.; Li, Y.-P.; Ji, W.-J.; Wang, K.; Qi, H.; Chou, P.-T.; Zhang, M.-M.; Bian, H.; Zhai, Q.-G., A New Molecular Recognition Concept: Multiple Hydrogen Bonds and Their Optically Triggered Proton Transfer in Confined Metal–Organic Frameworks for Superior Sensing Element. *ACS Appl. Mater. Interfaces* **2021,** *13* (19), 22457-22465.
6. Jayaramulu, K.; Kanoo, P.; George, S. J.; Maji, T. K., Tunable emission from a porous metal–organic framework by employing an excited-state intramolecular proton transfer responsive ligand. *Chem. Commun.* **2010,** *46* (42), 7906-7908.
7. Othong, J.; Boonmak, J.; Kielar, F.; Youngme, S., Dual Function Based on Switchable Colorimetric Luminescence for Water and Temperature Sensing in Two-Dimensional Metal–Organic Framework Nanosheets. *ACS Appl. Mater. Interfaces* **2020,** *12* (37), 41776-41784.
8. Yin, H.; Li, H.; Xia, G.; Ruan, C.; Shi, Y.; Wang, H.; Jin, M.; Ding, D., A novel non-fluorescent excited state intramolecular proton transfer phenomenon induced by intramolecular hydrogen bonds: an experimental and theoretical investigation. *Scientific Reports* **2016,** *6* (1), 19774.
9. Alarcos, N.; Gutiérrez, M.; Liras, M.; Sánchez, F.; Douhal, A., From intra- to inter-molecular hydrogen bonds with the surroundings: steady-state and time-resolved behaviours. *Photochemical & Photobiological Sciences* **2015,** *14* (7), 1306-1318.
10. Gutierrez, M.; Alarcos, N.; Liras, M.; Sánchez, F.; Douhal, A., Switching to a Reversible Proton Motion in a Charge-Transferred Dye. *J. Phys. Chem. B* **2015,** *119* (2), 552-562.
